# Alteration of rhesus macaque serum N-glycome during infection with the human parasitic filarial nematode *Brugia malayi*

**DOI:** 10.1038/s41598-022-19964-1

**Published:** 2022-09-21

**Authors:** Laudine M. C. Petralia, Esrath Santha, Anna-Janina Behrens, D. Linh Nguyen, Mehul B. Ganatra, Christopher H. Taron, Vishal Khatri, Ramaswamy Kalyanasundaram, Angela van Diepen, Cornelis H. Hokke, Jeremy M. Foster

**Affiliations:** 1 Division of Protein Expression and Modification, New England Biolabs, Ipswich, MA 01938 USA; 2 Department of Parasitology, Center of Infectious Diseases, Leiden University Medical Center, 2333 ZA Leiden, The Netherlands; 3 Department of Biomedical Sciences, University of Illinois College of Medicine at Rockford, Rockford, IL USA

**Keywords:** Carbohydrates, Glycomics, Mass spectrometry, Glycobiology, Post-translational modifications, Infectious diseases, Glycosylation, Diagnostic markers, Infectious diseases

## Abstract

Serum N-glycan profiling studies during the past decades have shown robust associations between N-glycan changes and various biological conditions, including infections, in humans. Similar studies are scarcer for other mammals, despite the tremendous potential of serum N-glycans as biomarkers for infectious diseases in animal models of human disease and in the veterinary context. To expand the knowledge of serum N-glycan profiles in important mammalian model systems, in this study, we combined MALDI-TOF-MS analysis and HILIC-UPLC profiling of released N-glycans together with glycosidase treatments to characterize the glycan structures present in rhesus macaque serum. We used this baseline to monitor changes in serum N-glycans during infection with *Brugia malayi*, a parasitic nematode of humans responsible for lymphatic filariasis, in a longitudinal cohort of infected rhesus macaques. Alterations of the HILIC-UPLC profile, notably of abundant structures, became evident as early as 5 weeks post-infection. Given its prominent role in the immune response, contribution of immunoglobulin G to serum N-glycans was investigated. Finally, comparison with similar N-glycan profiling performed during infection with the dog heartworm *Dirofilaria immitis* suggests that many changes observed in rhesus macaque serum N-glycans are specific for lymphatic filariasis.

## Introduction

Glycosylation is the most highly prevalent and structurally diverse post-translational modification of serum proteins. With the exception of albumin, all abundant proteins present in serum are glycosylated^1^, with the glycan moieties imparting a plethora of biological functions^2^. Glycosylation and variation thereof are determined by many factors such as glycosyltransferase gene expression levels, availability of nucleotide sugar donors, transit time through the endoplasmic reticulum and Golgi, and accessibility of each N-linked glycosylation site^1^. However, extensive characterization of N-linked glycosylation profiles of human serum by mass spectrometry (MS) and ultraperformance liquid chromatography (UPLC)^1,3–6^ have demonstrated the serum N-glycan profile of individuals to be generally highly stable^7^, although influenced by phenomena such as gender, ethnicity and aging^8,9^. Similarly, many studies involving multiple patient serum samples, have correlated specific changes in the type or abundance of serum N-glycans with different pathological conditions. As such, glycoprofiling for diagnostic serum glycan biomarkers has become an intense research focus for numerous diseases, most notably various cancers^10–13^, but extending to other conditions including cardiovascular disease and diabetes^14^, rheumatoid arthritis^15^, Crohn’s disease and ulcerative colitis^16^, congenital diseases^17,18^, and liver fibrosis^19^. In addition, subtle changes in the glycosylation of immunoglobulin G (IgG), the most abundant serum glycoprotein, can dramatically affect its ability to elicit effector functions^20–22^. Since IgG is readily purified from serum, it has also been subjected to detailed glycoprofiling^23–27^ and IgG N-glycans have proven effective biomarkers for a plethora of disease states^22^. Surprisingly, only a few studies have examined the IgG glycoprofiles from non-human species, yet these have revealed striking species-specific differences in both the nature and abundance of the appended N-glycans^28–30^. An even sparser literature exists on glycoprofiling of whole serum from other mammals^31,32^, despite the fact that many species have huge veterinary or agricultural value or serve as animal models in biomedical research.

Rhesus macaques (*Macaca mulatta*) have a rich history of use as a non-human primate model in multiple areas of biomedical research including successful vaccine development (*e.g.* smallpox and polio), evaluation of monoclonal antibody therapies for various conditions and infectious diseases, as well as drug discovery^33,34^. However, extrapolation of preclinical findings from macaques to humans can be compromised when subtle differences in biology exist. For example, interspecies differences between immunoglobulins and their Fc receptors raise questions about the suitability of macaques for development of human antibody therapies and vaccines, and the extent to which data from animal trials can be translated clinically^33,35,36^. Nonetheless, as of today, studies of rhesus macaque glycoconjugates have been limited to IgG N-glycans^28,37^.

One of many human diseases for which rhesus macaque have been used as an animal model is lymphatic filariasis (LF)^38,39^, a chronic, debilitating neglected tropical disease caused by several species of filarial nematodes. *Wuchereria bancrofti* (*W. bancrofti*), *Brugia malayi* (*B. malayi*) and *Brugia timori* (*B. timori*) are responsible for LF in humans. They infect over 50 million people worldwide, with about 36 million people showing the severe clinical pathologies of elephantiasis and hydrocele^40^. Adult worms live in the lymphatic system for up to 10 years where ovoviviparous females produce millions of microfilarial larvae that enter the peripheral circulation and can be acquired by blood-feeding mosquitos. Diagnosis is predominantly through cumbersome microscopic detection of microfilariae in blood or using the more convenient immunochromatographic strip tests, although limitations of these tests have also been reported^41,42^. Disease control is through vector control strategies and through repeated mass drug administration to endemic communities, which is not ideal due to the limited effect of the few available drugs on the long-lived adult worms, suboptimal treatment responses and contraindication in certain geographic areas where *Loa loa* is co-endemic^43–45^. Thus, further research into novel drug targets and diagnosis tools is needed to reach the World Health Organization elimination target as part of the NTD roadmap for 2030^46^. Most research directed at developing a vaccine, new therapies or diagnostics for LF employ *B. malayi* since this is the only filarial nematode of humans that can be readily maintained in laboratory animals (Mongolian gerbil) as well as cats and primates. Rhesus macaques, in particular have shown high similarities with humans in terms of lymphatic pathology and immune responses^38,39^. However, application of serum or IgG glycoprofiling for infectious diseases has focused mostly on infection with viral or bacterial pathogens^22,47^ with very few studies addressing infections with eukaryotic parasites such as protozoa^48–50^ or metazoan flatworms and nematodes^48,51,52^.

The present study describes a comprehensive characterization of N-glycans from rhesus macaque whole serum and purified IgG using matrix-assisted laser desorption/ionization time of flight mass spectrometry (MALDI-TOF-MS) in combination with orthogonal glycan sequencing techniques. We also present the N-glycan profiles of both serum and IgG of healthy rhesus macaques using hydrophilic interaction chromatography with ultra-performance liquid chromatography (HILIC-UPLC) coupled with fluorescence detection and MS. We used healthy macaque N-glycan profiles as a baseline for monitoring glycosylation changes in a longitudinal cohort of monkeys infected with the human filarial nematode *B. malayi,* that ranges from pre-infection through to establishment of mature infection with adult worms and microfilarial production. We report significant temporal changes in the relative abundance of individual glycan structures as well as classes of N-glycan structures (*e.g.* galactosylation and sialylation) that arise due to parasitic infection. These glycan profile changes expand our knowledge of the host response to filarial nematode infection and could provide insights into glycobiomarkers for LF infection in humans.

## Results

### MALDI-TOF-MS and HILIC-UPLC based characterization of rhesus macaque serum and IgG

**Figure 1:**
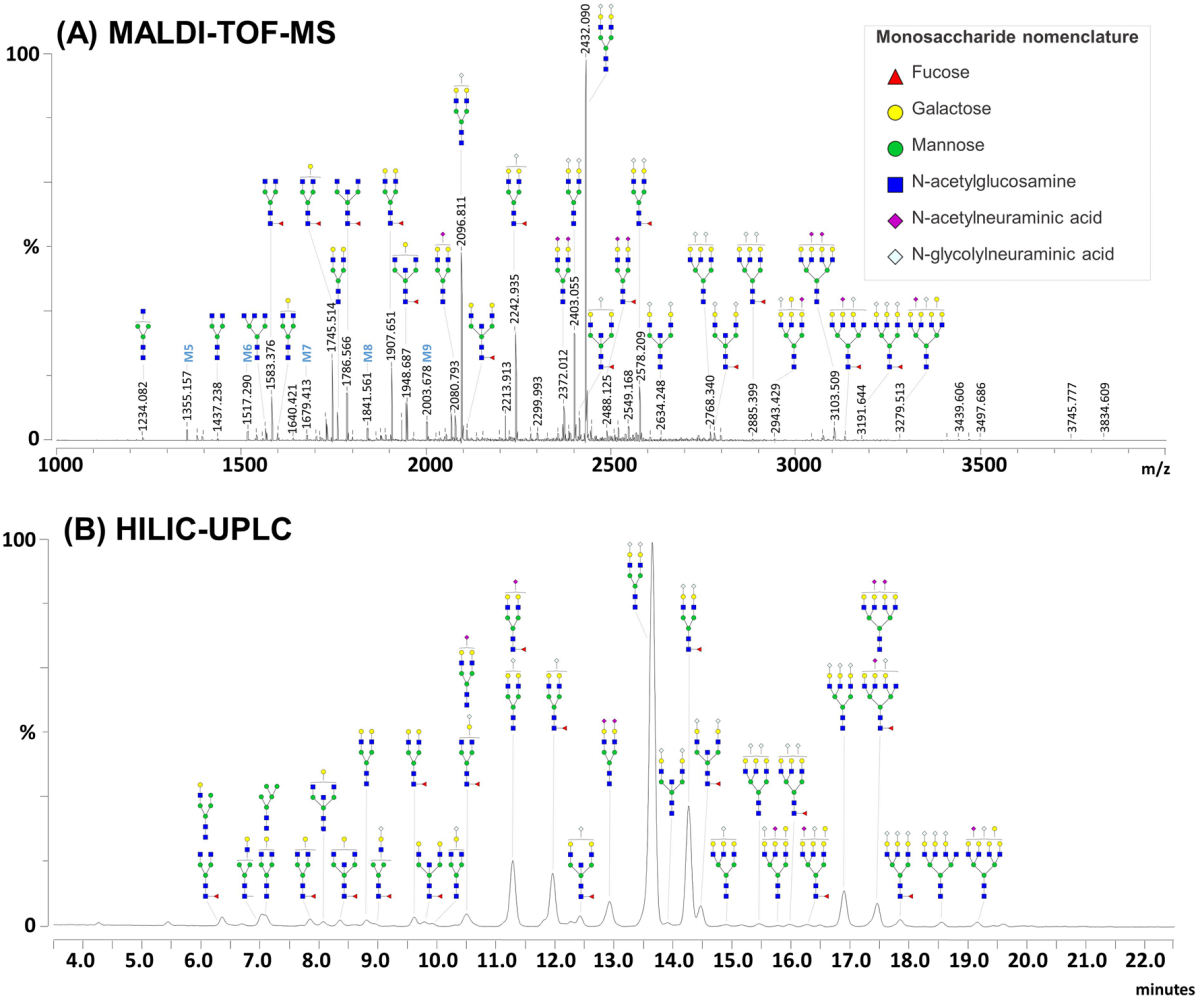
MALDI-TOF-MS analysis and HILIC-UPLC profiling of rhesus macaque serum N-glycans. PNGase F-released glycans from rhesus macaque serum glycoproteins were labeled with 2-AA and analyzed by MALDI-TOF-MS (**A**) or labeled with procainamide and analyzed using HILIC-UPLC (**B**). N-glycans were subjected to linkage-specific derivatization of the sialic acids by ethyl esterification prior to labeling with 2-AA for MALDI-TOF-MS. MALDI-TOF-MS signals were measured in negative ion reflectron mode and are labeled with monoisotopic masses (see Supplementary Table S1 and Fig. S1 online). N-glycan structures were assigned by combining data from both approaches and by using glycosidase digestions (see Supplementary Figs. S2 and S3 online). Proposed structures resulting from this analysis are depicted for selected peaks using the Consortium for Functional Glycomics (CFG) nomenclature (see symbol key inset). M5 to M9 designate mannosylated N-glycans with 5 to 9 mannose residues.

We characterized the N-glycans isolated from total serum glycoproteins of a pool of seven rhesus macaques and those from purified IgG using a combination of techniques including MALDI-TOF-MS and HILIC-UPLC. First, N-glycans were released from rhesus macaque serum glycoproteins, labeled with 2-AA and analyzed by MALDI-TOF-MS. This allowed the initial identification of 45 different serum N-glycan structures (Supplementary Fig. S1A online). An additional 38 sialylated N-glycan structures, mostly in the higher mass range, were detected with a similar analysis including a linkage-specific derivatization and stabilization of sialic acid using ethyl esterification^53,54^ (Fig. 1A and Supplementary Fig. S1B online). In total, 83 different N-glycan structures could be characterized. This number included many isomeric forms differing only by the linkages of the terminal sialic acids, as revealed using the specific derivatization that permits discrimination of α (2–3) and α (2–6) linkages. These 83 structures were represented by 65 different N-glycan compositions. Structural assignment was performed by digesting sequentially and individually the rhesus macaque serum N-glycans using the broad Neuraminidase A, Fucosidase O, β1–4 Galactosidase S and β-N-Acetylglucosaminidase (Supplementary Figs. S2 and S3 online). Using these enzymes sequentially, we reduced the N-glycans to their tri-mannosylated core and demonstrated the presence of a large variety of structures ranging from short, truncated N-glycans to multi-antennary complex N-glycans. Structures with up to four antennae (A1 to A4) were confirmed upon digestion with a mix of Neuraminidase A, Fucosidase O and β1–4 Galactosidase S. These antennae were extended with β-linked galactoses and sialic acids (Supplementary Fig. S2A online).

In parallel, we used a previously validated HILIC-UPLC profiling workflow to analyze PNGase F-released and procainamide-labeled N-glycans^32^ (Fig. 1B). Forty-three distinct integratable peaks (referred to as “glycopeaks” in the following sections) were identified and correlated with the serum profile (Supplementary Fig. S1C online). N-glycan structures comprising the glycopeaks were characterized using MS data obtained from the QDa mass detector linked to the UPLC instrument (see Methods) in combination with glycosidase digestions (Supplementary Fig. S2B online). Data from these orthogonal approaches were highly complementary, with only two minor glycopeaks from the HILIC-UPLC spectra not matching any MALDI-TOF-MS data. Similarly, 11 structures detected by MALDI-TOF-MS (mostly minor ions generated by short, truncated or high-mannose N-glycans) were not observed in HILIC-UPLC profiles.

Peak areas were quantified from HILIC-UPLC spectra and the relative abundance of each glycopeak compared to the total peak area of each spectrum. The most abundant N-glycan observed in rhesus macaque serum was biantennary A2G2S_Gc_2 which accounted for almost 48% of the total N-glycan pool. It was followed by the core-fucosylated version of the same structure (F(6)A2G2S_Gc_2) which accounted for over 10% (Supplementary Table S1 online). Due to the instability and ionization bias experienced by sialylated glycan species during MALDI-TOF-MS analysis^53–55^, the major ion in the MALDI-TOF-MS spectra of underivatized N-glycans corresponded to A2G2S_Gc_1 (*m/*z 2067.927 [M–H]^−^) (Supplementary Fig. S1 online). This was corrected using the linkage-specific derivatization method, where A2G2S_Gc_2 (2432.090 [M–H]^−^) appeared as the most abundant ion species. Notably, this analysis allowed for identification of *N-*acetylneuraminic acid (Neu5Ac) in many N-glycans (*e.g. m/z* 2372.012, 2488.125, 3103.509 [M–H]^−^, Fig. 1), although this form of sialic acid was less prominent than *N*-glycolylneuraminic acid (Neu5Gc). Using HILIC-UPLC, we estimated the proportion of N-glycans carrying Neu5Ac to be ~ 6% of the total glycan pool while 81% was sialylated with Neu5Gc. About 4% were complex N-glycans with high *m/z* (2800–4000 range) and late HILIC-UPLC retention times that possessed both sialic acid forms (*e.g. m/z* 3103.509, 3279.513 [M–H]^−^, and glycopeaks 35 at 17,4 min and 38 at 19,1 min, Fig. 1).

In addition to the sequential glycosidase digestion panels (Supplementary Fig. S2 online), complementary digestions with individual enzymes were also performed (Supplementary Fig. S3 online) to confirm serum N-glycan structural assignment. Notably, comparison of digestions with the specific α2–3 Neuraminidase S with broad-specificity α2–3,6,8,9 Neuraminidase A using HILIC-UPLC analysis showed that although the majority of the sialic acids were α2–6 linked, a significant proportion were α2–3 linked, supporting the assignments made by MALDI-TOF-MS analysis with sialic acid linkage-specific derivatization. Thus, by combining the glycosidase digestion results with HILIC-UPLC and MALDI-TOF-MS analysis, a comprehensive structural assignment of rhesus macaque serum N-glycans was obtained (Fig. 1). Detailed structural assignments and MALDI spectra annotated with glycopeak numbering are presented in Supplementary Fig. S1 and Table S1 online.

**Figure 2:**
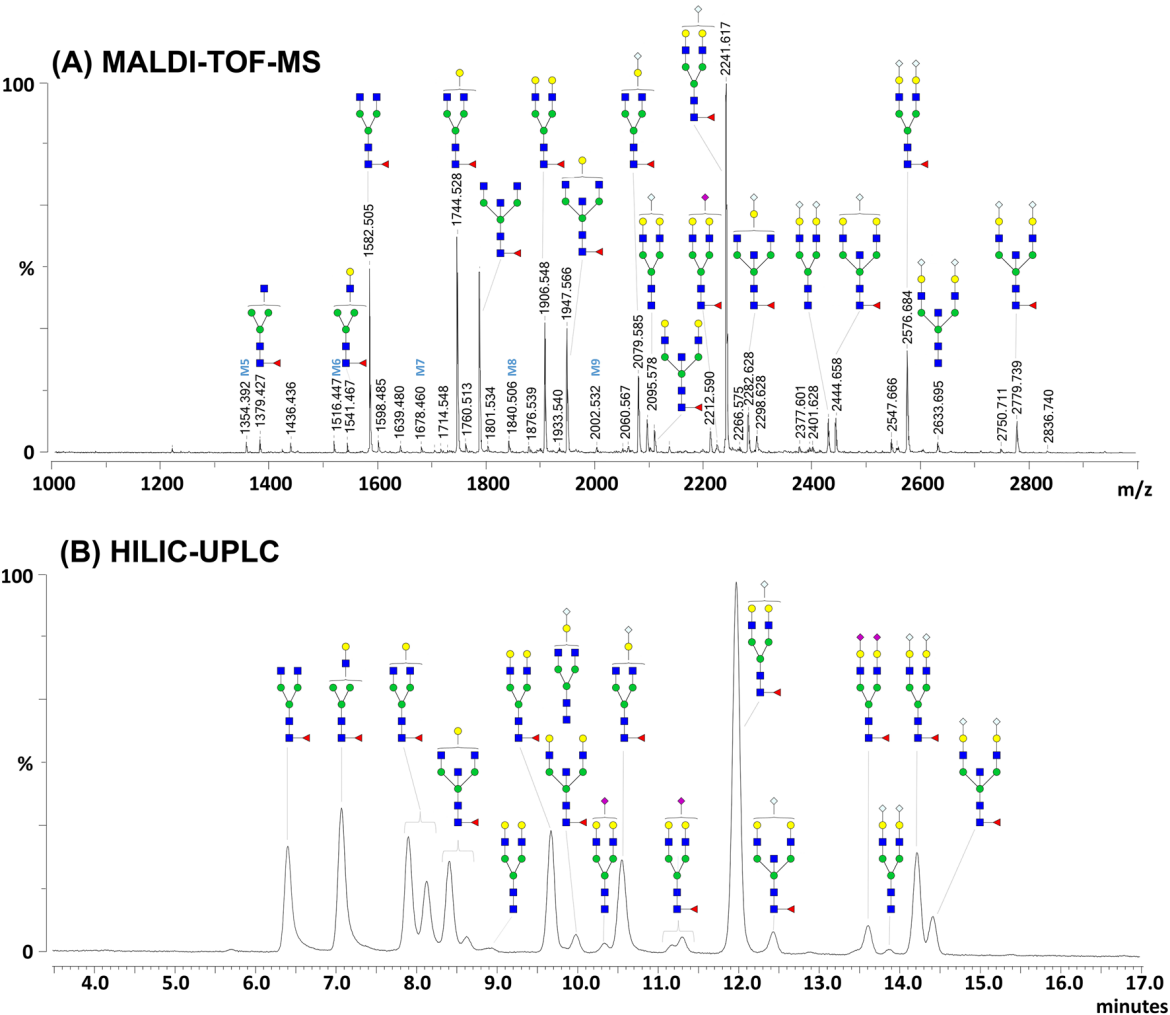
MALDI-TOF-MS analysis and HILIC-UPLC profiling of rhesus macaque IgG N-glycans. PNGase F-released glycans from purified rhesus macaque IgG were labeled with 2-AA and analyzed by MALDI-TOF-MS (**A**) or labeled with procainamide and analyzed using HILIC-UPLC (**B**). N-glycans were subjected to linkage-specific derivatization of the sialic acids by ethyl esterification prior labeling with 2-AA for MALDI-TOF-MS. MALDI-TOF-MS signals were measured in negative ion reflectron mode and are labeled with monoisotopic masses (see Supplementary Table S2 and Fig. S4 online). N-glycan structures were assigned by combining data from both approaches and by using glycosidase digestions as well as MALDI-TOF-MS/MS (see Supplementary Figs. S5 and S6 online). Proposed structures resulting from this analysis are depicted using the CFG nomenclature (see symbol key inset in Fig. 1). M5 to M9 designate mannosylated N-glycans with 5 to 9 mannose residues.

An identical analysis was performed on IgG purified from the rhesus macaque pool of sera (Fig. 2). Using MALDI-TOF-MS, 26 N-glycan structures were identified that were further extended to 43 with the additional step of ethyl esterification of sialic acids (Supplementary Fig. S4 online). In addition, 19 glycopeaks were observed in the HILIC-UPLC profile that correlate with 30 of the N-glycans identified using MALDI-TOF-MS, with the exception of glycopeak #16 (Supplementary Table S2 online). This resulted in a total of 13 N-glycan structures being identified by MALDI-TOF-MS analysis that were not detected by HILIC-UPLC profiling (due to their lower abundance).

These N-glycans were further characterized using sequential and individual glycosidase digestion. This analysis revealed that IgG N-glycans were comprised of structures bearing terminal sialic acids, terminal β-linked galactoses, α-linked fucoses, or were mannosylated structures (Supplementary Figs. S5 and S6 online). Finally, tandem MS using MALDI-TOF (MALDI-TOF-MS/MS), confirmed the presence of bisecting GlcNAcs on some structures, consistent with prior observations on rhesus macaque IgG N-glycans^28,37^. Fragmentation profiles of selected ions with theoretical *m/z* 1436.53 and 1639.61 [M–H]^−^ from IgG N-glycans both indicate the presence of bisecting GlcNAc (Supplementary Fig. S6B online).

### N-glycan profiles of serum and IgG from human and rhesus macaques exhibit differences

**Figure 3:**
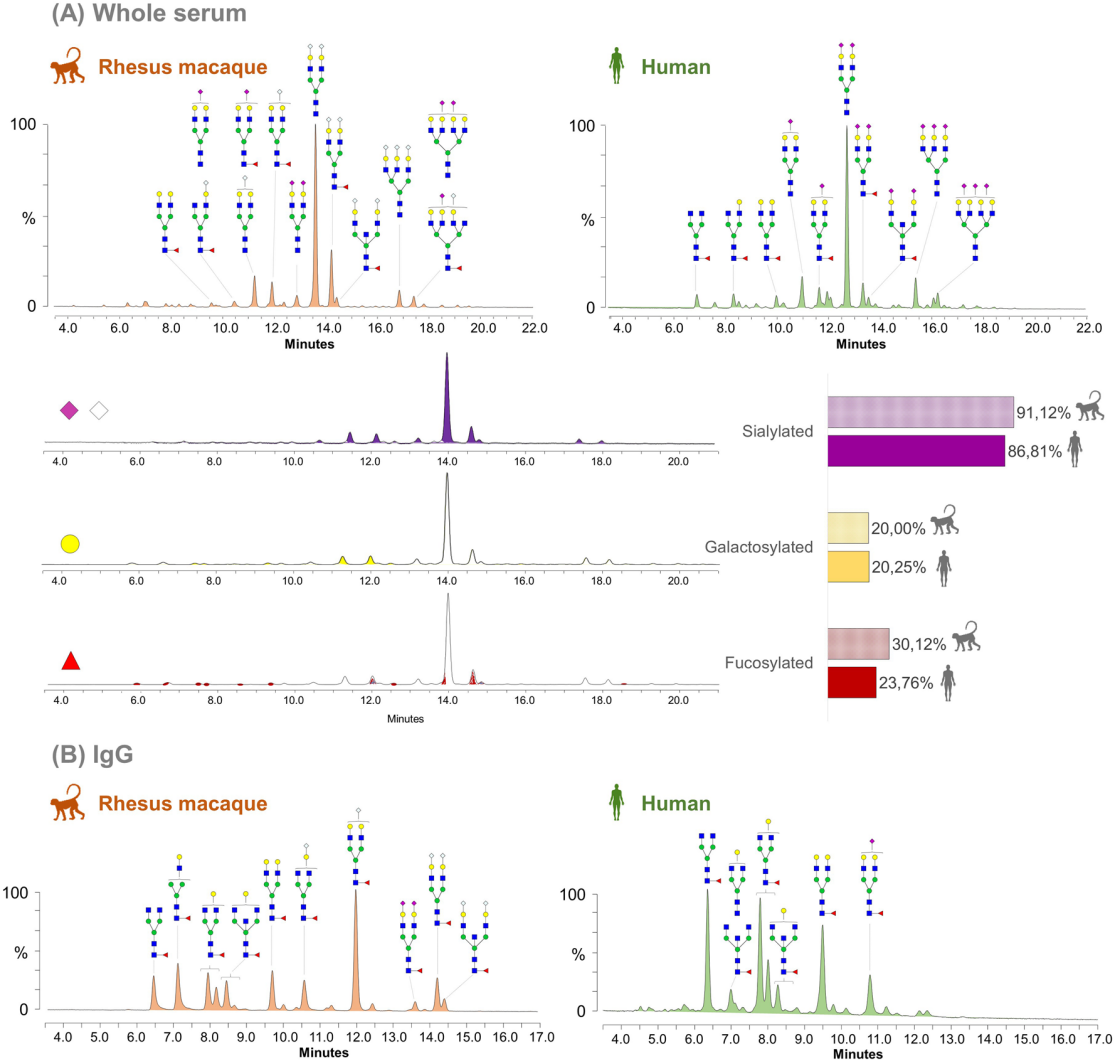
Comparison of rhesus macaque and human HILIC-UPLC profiles of serum (**A**) and IgG (**B**) N-glycans. HILIC-UPLC spectra of rhesus macaque (left) and human (right) are shown for both serum (**A**) and IgG (**B**). N-glycan structures found in the 10 major glycopeaks (based on peak areas) are depicted using the CFG nomenclature (see inset in Fig. 1). Quantification of glycan classes in rhesus macaque serum was determined before and after digestion with α2-3,6,8,9 Neuraminidase A for sialylated structures (purple and grey diamonds), β1-4 Galactosidase S for structures carrying terminal galactose (yellow circle) and with Fucosidase O for core-fucosylated structure (red triangle). Results were compared with similar quantification data conducted previously on human serum^32^ and are shown on the bar chart on the right.

Exoglycosidase digestions combined with HILIC-UPLC analysis permitted quantification of the following N-glycan categories in rhesus serum: galactosylated (N-glycans bearing at least one terminal unsubstituted galactose), fucosylated (fucose-containing N-glycans), sialylated (N-glycans having at least one antenna terminated by sialic acid) and mannosylated (oligomannosidic or hybrid N-glycans). Analysis of human serum N-glycans was previously conducted using the same workflow^32^, allowing a direct comparison. Most rhesus macaque serum N-glycans are sialylated, with over 91% of the UHPLC profile area being sensitive to α2–3,6,8,9 Neuraminidase A treatment (Fig. 3A). This percentage appeared higher than observed for humans (~ 87%). The majority of the sialic acids in rhesus macaque serum N-glycans are Neu5Gc. This is consistent with the presence of a putative functional CMP-N-acetylneuraminic acid hydroxylase (CMAH) gene—the only enzyme capable of synthesizing Neu5Gc by hydroxylation of Neu5Ac^56–58^—in rhesus macaques^59^. This gene is deactivated in a number of lineages including that leading to humans^60^. Thus, while the major structure in human is A2G2S2, the major structure in rhesus macaques is A2G2S_Gc_2. Using Fucosidase O digestion, we determined that around 30% of the total N-glycan serum pool is fucosylated in rhesus macaques while it was found to be slightly less than 24% in humans. The abundance of FA2G2S_Gc_2 in rhesus macaques largely contributes to this overall higher fucosylation, since the ratio between the A2G2S_Gc_2 and its fucosylated variant (FA2G2S_Gc_2) in rhesus macaque is considerably lower than between A2G2S2 and FA2G2S2 in human. We also noted a smaller amount of agalactosylated N-glycans in rhesus macaque serum compared to human serum, which was particularly evident for the F(6)A2-containing glycopeak, while the proportion of galactosylated N-glycans was similar in both species at 20% of the total N-glycan pool. Digestions with β1–4 Galactosidase showed that all galactoses were β1–4 linked and this was confirmed by the absence of sensitivity to the broad-specificity α1–3,4,6 Galactosidase (Supplementary Fig. S3 online), consistent with the absence of α1,3 galactosylation in the common ancestor of old world primates due to the loss of α1,3 galactosyltransferase^61^. Finally, a small portion (around 3%) of mannosidic and hybrid N-glycans, which have also been found in humans, was revealed using Endoglycosidase H (Supplementary Fig. S3 online).

In accordance with previous studies^28,37^, we also found the IgG N-glycans of rhesus macaque to be substantially different from human IgG (Fig. 3B). Although IgG profiles from both species share many, largely core-fucosylated, N-glycan structures, the relative proportion of these N-glycans differed considerably. Indeed, the rhesus macaque IgG profile is dominated by the N-glycan structure F(6)A2G2S_Gc_1, while the agalactosylated (F(6)A2) and galactosylated (F(6)A1-2G1-2) structures, which constitute the majority of human IgG N-glycosylation, appear in markedly lower amounts on rhesus macaque IgG. Thus, rhesus macaque IgG exhibits a significantly higher abundance of larger sialylated N-glycans compared to human, which differentially impacts the contribution of IgG N-glycans to the total serum N-glycoprofile. Indeed, in human, IgG N-glycans are nearly all smaller than A2G2S2 and, thus, do not contribute to the most abundant complex N-glycan structures^1^, while in rhesus macaque, IgG N-glycans contribute to FA2G2S_Gc_2 and FA2(B)G2S_Gc_2 (glycopeaks #25 and 26), two major sialylated N-glycans of the HILIC-UPLC serum profile. As for serum N-glycans, the vast majority of the IgG sialic acids were Neu5Gc. However, we detected a minor amount of the Neu5Ac form of sialic acid in several glycopeaks (Fig. 2 and Supplementary Table S2 online) which has not been reported previously for rhesus macaques^28,37^.

Finally, we also found bisecting GlcNAc which is a common feature in both species, but has been determined to be more prominent in rhesus macaques compared to humans^37^. Although we did not perform any quantitative analysis of bisecting GlcNAc-containing N-glycans, we detected them in a significant amount on rhesus macaque IgG (Supplementary Fig. S5 online). Presence of bisecting GlcNAc explains the incomplete digestion of the structure FA2B (*m/z* 1639.506 [M–H]^−^) using N-acetylglucosaminidase (residual peak *m/z* 1395.347 [M–H]^−^).

### Changes in serum N-glycosylation during the course of *B. malayi* infection

**Figure 4:**
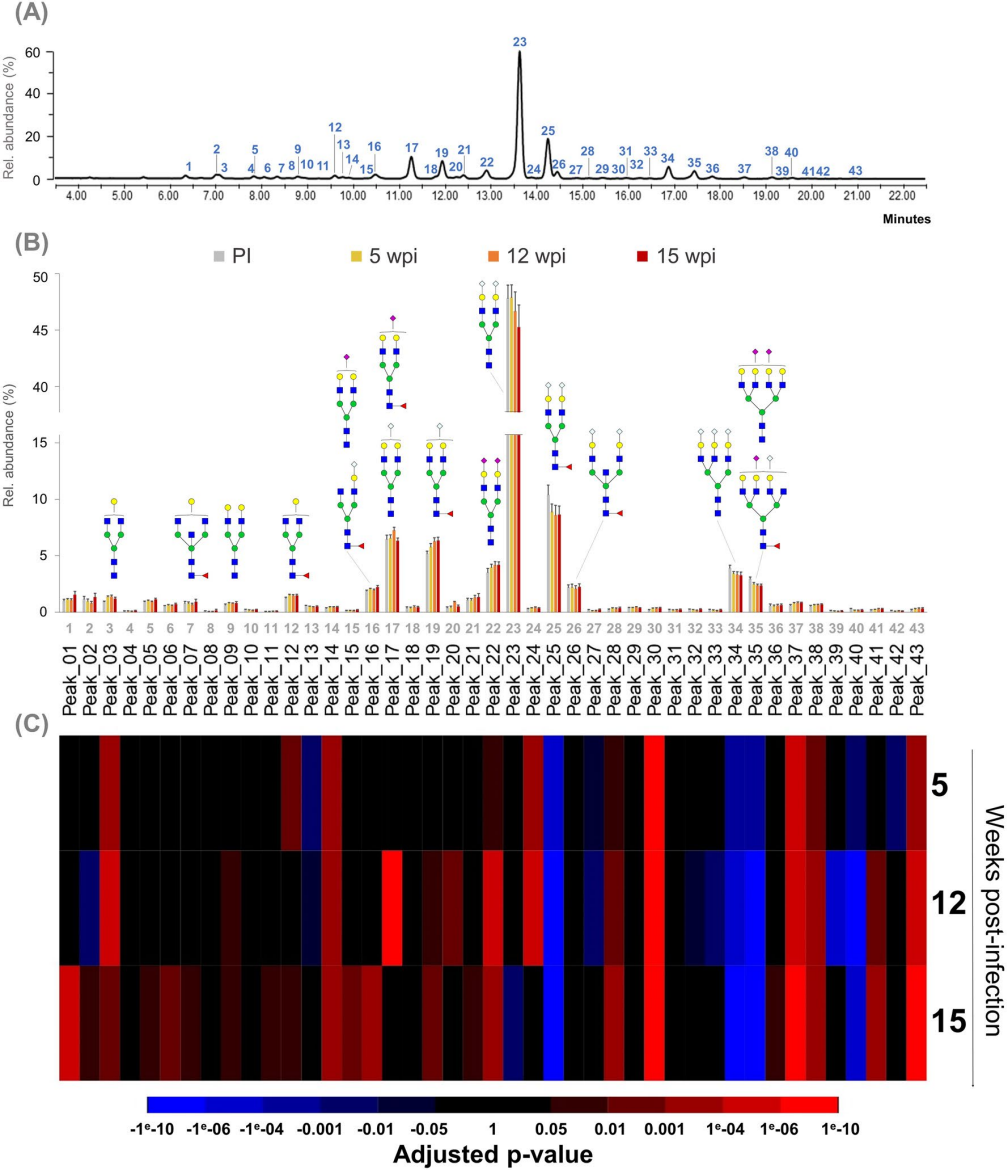
Changes in HILIC-UPLC profile of rhesus macaques serum N-glycans during infection with *B. malayi*. Quantification of individual peaks derived from HILIC-UPLC spectra was performed for 4 different animals at 4 different time-points namely pre-infection (PI), 5 weeks post infection (wpi), 12 wpi and 15 wpi (see Supplementary Table S4A online). The HILIC-UPLC traces obtained at pre-infection for the 4 animals are overlaid in (**A**), and glycopeak numbering is indicated. Individual glycopeak areas were integrated and quantified at each of the time-points. Average of glycopeak relative abundance (Rel. abundance) for the 4 animals are shown on the bar graph (**B**) with standard error bars. The x-axis displays the peak numbers corresponding to the HILIC-UPLC profile. Using linear mixed-effect models, changes in the abundance of serum *N*-glycans during infection were assessed and results of the statistical analysis for each glycopeak are presented in the heatmap (**C**). Decrease in abundance (blue) and increase in abundance (red) is relative to baseline (pre-infection) for each time point (see color key). Adjusted *p*-values can be found in Supplementary Table S4B online. Selected glycan structures are represented using the CFG nomenclature (inset in Fig. 1).

To study if infection with *B. malayi* affects serum N-glycosylation, we used the HILIC-UPLC workflow to monitor the profiles from a longitudinal set of rhesus serum samples over the course of their infection with this filarial nematode. Firstly, serum samples from 7 different healthy animals were analyzed separately with technical duplicates and yielded highly similar HILIC-UPLC profiles resulting in a stable baseline for the serum N-glycan profile of healthy rhesus macaques (Supplementary Fig. S7 and Table S4A online). Secondly, serum was sampled from four of these rhesus macaques at 5, 12 and 15 weeks post-infection (wpi) with *B. malayi* infective larvae (Methods section and Supplementary Table S3 online). Again, serum N-glycans were analyzed using HILIC-UPLC for each individual animal, at each time-point, with technical duplicates (Supplementary Table S4A online). To study the alteration of the N-glycan serum profile during the course of *B. malayi* infection, we used statistical analysis based on linear-mixed effect models^62^, to compare the HILIC-UPLC profiles of individual animals from the longitudinal cohort at 5, 12 and 15 wpi to their corresponding pre-infection ones. Changes arose as early as 5 wpi when 17 glycopeaks showed significant differences in their relative peak area (in percentages) between infected and baseline time-points (adjusted *p*-value < 0.05). Alteration of serum N-glycosylation increased as the infection progressed, with 24 glycopeaks showing statistically significant changes in abundance at 12 wpi and 27 glycopeaks at 15 wpi. Many altered glycopeaks were abundant ones in the HILIC-UPLC profile with, for instance, the major glycopeak (#23 in the HILIC-UHPLC profile) that significantly decreased at 15 wpi or glycopeak #25 that showed a significant reduction as early as 5 wpi and continued until 15 wpi when compared to baseline. Interestingly, many of the early changes—either a decrease or increase in relative abundance—lasted up to 15 wpi as this was observed for 12 out of the 17 glycopeaks (Fig. 4 and Supplementary Table S4B online). In addition, some noticeable changes became evident at 15 wpi, with a clear increase for many shorter glycan structures that appear early in the HILIC-UPLC profile (*e.g.* glycopeak #1, 2, 3, 5, 6, 7, 9 11, 12, 15, 16, 20) and a significant decrease for the most abundant serum N-glycan (glycopeak #23). Overall, the directionality of changes appeared to be consistent over the course of the infection, since only glycopeak #2 fluctuated (decrease at 12 wpi and increase at 15 wpi), while the relative abundance of all other glycopeaks that showed changes were consistently increased or decreased throughout the longitudinal study.

We also analyzed N-glycosylation of purified IgG from serum of all 4 infected monkeys at each time-point. In contrast, total IgG glycosylation in infected rhesus macaques did not show any significant changes in N-glycosylation over the course of infection (data not shown).

**Figure 5:**
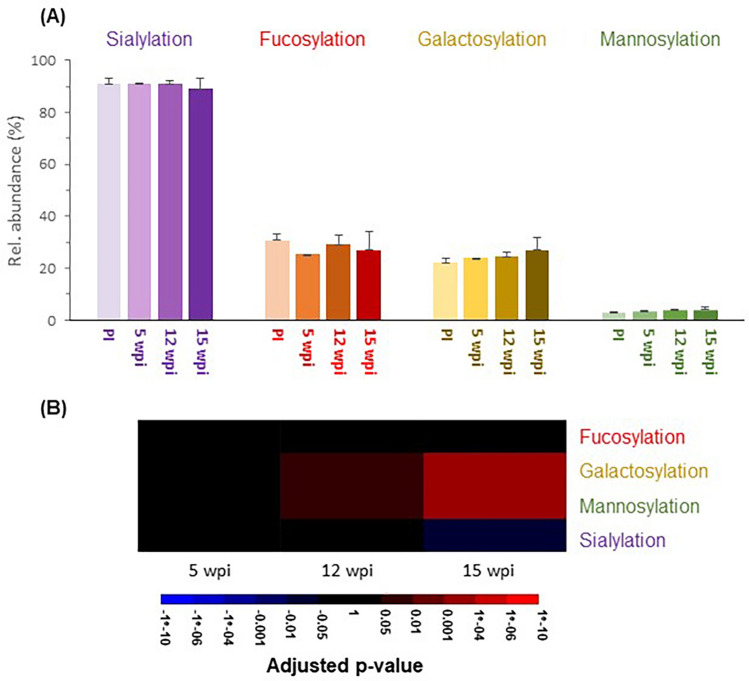
Changes in the major classes of rhesus macaque serum N-glycans during the course of infection with *B. malayi*. N-glycan content of each glycopeak in the UPLC profile was structurally characterized using a combination of UPLC-profiling, MALDI-TOF-MS and glycosidase digestions. Based on their glycan content, glycopeaks belonged to the following glycan classes: sialylated (purple), fucosylated (red), galactosylated (yellow) and/or mannosylated (green). Relative abundance (Rel. abundance) of each class of glycan was quantified by adding individual peaks areas belonging to the same class. This was done for each animal (n = 4) at each of 4 time-points: pre-infection (PI), 5 weeks post-infection (wpi), 12 wpi and 15 wpi. Results of this analysis are shown by the bar chart (**A**). The relative abundance of each glycan class was averaged for all animals and shown as percentages with standard deviation as error bars. Statistically significant changes in relative abundance of glycan classes in the course of the infection were estimated using linear mixed-effect models. Adjusted *p*-values can be found in Supplementary Table S5 online and were used to generate the heatmap (**B**). Blue color indicates a decrease in abundance and red an increase.

We next aimed to investigate trends within the main N-glycan classes present in rhesus macaque serum during infection. Structural assignment performed on the basis of exoglycosidase digestions allowed us to estimate the relative abundance of sialylated, fucosylated, galactosylated and mannosylated (oligomannosidic or hybrid) N-glycans at each time-point (Fig. 5 and Supplementary Table S5 online). Unlike changes in individual N-glycan structures, the first significant changes in these broad N-glycan classes appear only at 12 wpi with an increase in both galactosylation and mannosylation. However, at 15 wpi this increase in galactosylated and mannosylated N-glycans appears to become more substantial (with *p* values < 0.001) and is accompanied by a decrease in sialylated structures. This finding is in line with changes in individual glycans described above since structures eluting at the earliest retention times are mainly galactosylated and mannosylated. Thus, the increased abundance of these structures at 15 wpi and the decreased abundance of the major sialylated N-glycan likely explain the observed trends for the N-glycan classes.

**Figure 6:**
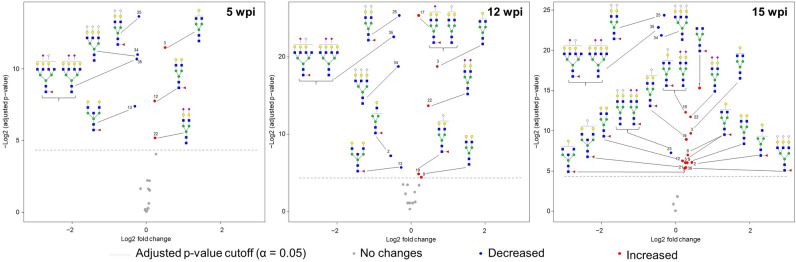
Changes in the major HILIC-UPLC glycopeaks of the rhesus macaque serum N-glycan profile upon infection with *B. malayi*. Glycopeaks from the HILIC-UPLC profiles of healthy rhesus macaque serum N-glycans with areas above 0.5% of the total profile area at baseline (pre-infection) were selected. Changes in the relative abundance of the 20 glycopeaks resulting from this selection were studied across infection by comparing the post-infections time-points: 5 (**A**), 12 (**B**) and 15 (**C**) weeks post-infection (wpi)—with pre-infection (baseline). Adjusted p-values were determined using linear-mixed effect models (Supplementary Table S4 online) and volcano plots were generated for data visualization. The X-axis of the plot corresponds to the log2 fold changes of glycan abundance and the y-axis to the negative log2 of the adjusted p-values. The horizontal dashed line represents the threshold of significance α = 0.05. All glycopeaks showing significant differences between baseline and infected time-points are indicated by a colored datapoint (red = increase, blue = decrease). Corresponding N-glycan structures are represented using the CFG nomenclature (see Fig. 1 inset), adjacent to the datapoint corresponding to each glycopeak, numbered according to elution in the HILIC-UPLC profile (Fig. 4.A and Supplementary Fig. S1 online).

Given that the main goal of performing such glycoprofiling studies is to identify potential glycan biomarkers of infection or disease, we decided to focus on changes in the most abundant N-glycan structures which constitute more promising targets for diagnostic application. Since we noticed a slightly higher technical variation for the smallest peaks in the profile and for those with the later retention times, we selected the 20 glycopeaks with areas larger than 0.5% of the total profile area and with a retention time less than 18 min in the HILIC-UPLC profile. The volcano plots summarizing the results of the statistical analysis performed on these peaks are shown in Fig. 6. As previously seen when studying changes for the overall HILIC-UPLC profile, the number of glycopeaks showing statistical differences compared to healthy serum increased during infection. Thus, in this glycopeak selection, 7 peaks are significantly altered at 5 wpi, 10 at 12 wpi and 17 peaks at 15 wpi. Moreover, this was accompanied by a noticeable increase in the negative log2 of the adjusted p-values. Interestingly, 6 out of the 7 glycopeaks showing differences as early as 5 wpi were either decreased (#13, 25, 34, 35) or increased (#3, 22) throughout the 3 time-points, supporting consideration of these glycan structures as potential biomarker candidates.

Finally, taking advantage of the use of the exact same workflow for both studies, we compared the changes observed in rhesus macaque N-glycans during infection with *B. malayi* with the changes in canine serum N-glycans during infection with the dog heartworm *Dirofilaria immitis* (*D. immitis*)^51^. We compared the alteration of serum N-glycosylation observed at 27 wpi for the dogs and at 15 wpi for the rhesus macaques since these time-points coincide with the microfilariaemic stage of infection for the animals in both studies. Both filarial infections impact the serum N-glycosylation and we focused on N-glycan structures that were identified in both dog and rhesus macaque serum (Supplementary Table S6 online). Some similarities could be observed, for instance the abundance of F(6)A2, F(6)A2G1, F(6)A2G2 and F(6)A2G2S_Gc_1 is increased in both infections, while abundance of F(6)A2G2S_Gc_2 decreased in both cases. However, several glycan structures were differentially impacted in both studies. As an example, abundance of A2G2S_Gc_2 decreased in rhesus macaque serum upon microfilariaemia while it increased in dog serum. Thus, we could not highlight a very clear common trend for both filarial infections and our data tends more toward a parasite- and species-specific alteration of the serum N-glycome.

## Discussion

In this study, we first conducted a comprehensive characterization of rhesus macaque serum N-glycans. We used MALDI-TOF-MS analysis and HILIC-UPLC profiling together with glycosidase-based glycan sequencing and available knowledge of mammalian glycosylation pathways^63^ to propose the most likely and complete structural assignment of serum and IgG N-glycans (Figs. 1, 2 and Supplementary Tables S1 and S2 online). This combination of technologies was highly complementary. Extreme sensitivity of the MALDI-TOF-MS analysis allowed a broad coverage of the serum N-glycome structures, while HILIC-UPLC profiling provided quantitative information. The addition of a linkage-specific derivatization of sialic acid was particularly helpful for the analysis of N-glycan structures containing these residues. Rhesus macaque serum, like human serum, appeared to be rich in complex, bi to tetra antennary, highly sialylated N-glycans. Neu5Gc, a sialic acid not occurring in humans due to the absence of an active CMAH gene, was the major form of sialic acid detected in rhesus macaque serum N-glycans, while Neu5Ac was also present in a lower proportion. Absence or presence of Neu5Gc is thought to affect a plethora of biological functions particularly immunity, with differences in susceptibility to pathogens and cell reactivity due for instance to changes in Siglec binding^64^ and it is not clear to what extent the effects of Neu5Gc in rhesus macaques mirror those of Neu5Ac in humans. In addition to this major difference between human and rhesus macaque serum N-glycosylation, elevated fucosylation levels were also observed in rhesus macaque.

Given its significant contribution to serum N-glycans and relevance in immunology, we also examined rhesus macaque IgG N-glycans. We observed features expected from the literature such as core-fucosylation of virtually every structure, the presence of Neu5Gc and bisecting GlcNAcs^28,37^. The latter is consistent with a gene homolog of the human beta-1,4-mannosyl-glycoprotein 4-beta-N-acetylglucosaminyltransferase known to exist in rhesus macaques (https://www.ncbi.nlm.nih.gov/, Gene ID: #703643). In addition, some truncated (*e.g.* FA2) and galactosylated N-glycans (*e.g.* FA2G1, FA2BG1, FA2G2), major features of human IgG N-glycans, were also detected in rhesus macaque IgG N-glycans. FA2G1 and FA2BG1 have been reported to be preferentially galactosylated on the α1–6 branch of the N-glycan core for both humans and rhesus macaques^28^. Our data corroborated this observation with the ratios of FA2G1 and FA2BG1 respective isomers being almost identical for rhesus macaques and humans (Fig. 3B) which might be indicative of a highly conserved β-1,4-galactosyltranseferase specificity and activity in both species. Despite these similarities with humans, the N-glycan profile of rhesus macaque IgG also showed major differences. In particular, although truncated and galactosylated structures were significantly represented, the major N-glycan species found on rhesus macaque IgG was FA2G2S_Gc_1 (HILIC-UPLC glycopeak #14, Supplementary Fig. S2A and Table S2 online). In addition, disialylated complex N-glycan structures FA2G2S2 (glycopeak #16), FA2G2S_Gc_2 (glycopeak #18) and FA2BG2S_Gc_2 (glycopeak #19) constitute a significant proportion of rhesus macaque IgG N-glycans. Altogether, rhesus macaque IgG has a significantly higher proportion of sialylated N-glycans than human IgG. Our results are mostly in line with the characterization of rhesus macaque IgG N-glycans conducted previously^28^ whereby FA2, FA2G1-2, FA2G1S_Gc_1, FA2G2S_Gc_1-2 were identified as major structures and FA2B, FA2BG1-2, FA2BG1S_Gc_1, FA2BG2S_Gc_1-2 were detected as well albeit in lower amounts. Differences between these findings, in particular the larger number of N-glycans identified in our analysis—can easily be explained by improvement of methods and MALDI-TOF-MS technologies over the past decades as well as the use of HILIC-UPLC for quantification in our study, which was not performed earlier^28^. Higher levels of sialylated N-glycans were measured for rhesus macaque IgG than for humans in the previous study, matching our observations. However, a significantly lower number of acidic sugars than neutral sugars was found, which appears somewhat contradictory to our results. In addition, only the Neu5Gc form of sialic acids was detected, while we identified 4 HILIC-UPLC glycopeaks and 6 MALDI-TOF-MS ions containing Neu5Ac (Supplementary Table S2 online). Improved analytics, especially the linkage-specific derivatization of sialic acids, can again easily explain this divergence particularly given the very low abundance of Neu5Ac.

The predominance of the Neu5Gc form of sialic acid in the rhesus macaque N-glycan IgG profile raises the question of possible biological implications and immune differences between rhesus macaques and humans. Interestingly, the importance of sialic acid on N-glycans of the IgG Fc region is unclear. Some studies have suggested that sialylation of Fc N-glycan is necessary for the IgG anti-inflammatory properties^65,66^ while more recent work showed that sialic acid does not affect recognition by Fc γ receptors (FcγRs) III^36,67,68^. Thus, despite major differences in IgG N-glycans, including the type of sialic acid, rhesus macaques might have Antibody-Dependent Cellular Toxicity mechanisms that are highly similar to humans. Presence or absence of core-fucosylation, in particular, has been shown to be one of the major mediators of functional changes in IgG for FcγR- and complement-mediated effector functions, together with terminal galactosylation^69,70^. Our analysis showed the presence of both N-glycan features on healthy rhesus macaque IgG, which appears in line with the aforementioned hypothesis and strengthens the idea of rhesus macaques as a valid animal model for translation to humans.

We believe that our study contributes to a better knowledge of rhesus macaque serum N-glycans which is highly relevant to many studies where they serve as a model for human diseases. Here, we defined the healthy macaque serum N-glycoprofile and used it as a baseline to study the changes in N-glycosylation during establishment of infection with the parasitic filarial nematode *B. malayi*. This longitudinal serum set did not yield striking visual changes within the serum N-glycan profiles such as those that have been observed in infection with the filarial nematode *D. immitis* in dogs^51^. Nonetheless, we highlighted many statistically significant changes between the various post infection time-points and the stable baseline defined by the uninfected profile (Fig. 4). When focusing on broad classes of glycans, we noted an overall increase of galactosylation and a reduction in sialylation that were similarly reported for *D. immitis* infections^51^. Interestingly, although higher degrees of fucosylation have previously been associated with various disease conditions^71–73^—including during infection with *D. immitis*^51^—we could not observe any clear and statistically significant trend regarding changes in the abundance of fucosylation during primate infection with *B. malayi* (Fig. 5).

At the individual N-glycan level, changes arise early, as soon as 5 wpi, when none of the animals was yet microfilariaemic (Supplementary Table S3 online). This is consistent with immune responses to parasite (glycan) antigens already occurring at 5 wpi^74^. Surely, given their significant contribution to serum N-glycans^1^, induction of Igs to *B. malayi* antigens (BmA) may affect the overall profile. In particular, specific IgM to parasite glycans has been observed to appear early during infection (at 5 wpi) and to decrease gradually thereafter^74^. This dynamic of early immune response might explain early changes in the N-glycoprofile (*e.g.* glycopeaks #12, 17, 24, Fig. 4). Nonetheless, most of the changes observed appeared to be “mono-directional” (either increase or decrease) from 5 to 15 wpi, which is an advantage for putative biomarkers. When focusing on the most abundant N-glycans (Fig. 6), structures that appear to decrease upon infection are mostly sialylated complex N-glycans while several galactosylated N-glycans increase in relative abundance. This result which was also reflected in the analysis of the changes in glycan classes (Fig. 5), could possibly be a consequence of an impact of LF on the sialyltransferase or CMAH activity. A critical question to further evaluate is whether those changes would be maintained on the longer term. Indeed, in serum of dogs infected with *D. immitis*, out of the 25 HILIC-UPLC N-glycan glycopeaks that were up or down-regulated after 27 weeks of infection—which coincided with the start of microfilaremia production—only 5 were still altered in chronically infected dogs (after 2.5 years of patent infection). Our sample set does not allow us to tackle this question so study of serum from chronically infected animals would be necessary. In addition, our study used a relatively small group of young macaques. Future studies should consider a larger cohort that includes a wider diversity of individuals to address the effect of natural phenomena, such as aging, that might affect the serum N-glycan profile^8,9^. Moreover, it would be important to study when the serum N-glycan profile returns to normal after anthelmintic treatment of LF—if it ever does.

The origins of the observed changes in serum N-glycosylation—resulting from changes in glycan microheterogeneity and/or serum protein composition/relative amount—are of major interest. In humans, over 15 different proteins are thought to contribute to the major N-glycan structure of total serum A2G2S2^1^, and we might expect something similar for A2G2S_Gc_2 in rhesus macaques. In this study, total IgG glycan microheterogeneity does not appear to be affected by LF unlike observations for many human diseases ranging from cancers to intestinal or liver conditions^73,75,76^. In addition, in both bancroftian LF^52,77^ and leishmaniasis^49^, differences in IgG Fc N-glycosylation have also been identified between different subpopulations of infected individuals (*i.e.* symptomatic or asymptomatic). Unfortunately, our dataset did not allow us to address this question for LF in rhesus macaques since all animals in the cohort were symptomatic (Supplementary Table S3 online). It would also be of interest to investigate N-glycans of *B. malayi* antigen-specific IgG. For many other infectious diseases, antigen-specific IgG Fc glycosylation has indeed shown robust glycosylation profiles between individuals, when total IgG would not, or have been associated with different degree of disease^47^.

The observed changes might however originate in part from increased levels of IgG in total serum, as observed in dog serum in response to infection with *D. immitis*. This would be consistent with the fact that many IgG glycopeaks appear to be upregulated at 15 wpi (HILIC-UPLC glycopeaks #1, 5, 6, 7, 9, 12, 14, 16, 19, 21). Nonetheless, this pattern is considerably less pronounced in our study than for *D. immitis* infection in dogs. Thus, it would need confirmation and cannot explain the totality of the changes we observed. Consequently, it would be of great interest to investigate a broader spectrum of serum glycoproteins to address the question of the origin of the observed N-glycan alterations. Glycoproteomic studies appear particularly promising in the search for biomarkers. For instance, alpha-1-acid glycoprotein (AGP), an acute phase glycoprotein in serum has been shown to present alterations of N-glycans specific for type-II diabetes or prostate cancer^78,79^. Moreover, multiplying and coupling a selection of diverse molecules (so called panel of biomarkers) has already given very positive results for early detection of hepatocellular carcinoma in humans^80,81^. This could be a strategy to consider here since the large number of altered N-glycan structures suggest an impact of LF on more than one serum glycoprotein. This is also consistent with observations of an increase in the circulating levels of various serum glycoproteins being associated with pathogenesis of disease in human LF^82^. In conclusion, we appreciate that the data presented here derive from a small cohort of animals, restricted to the disease establishment period and that further studies are needed to develop serum N-glycans as possible biomarkers for infection in humans. Nonetheless, we report a clear effect of disease establishment on the rhesus macaque serum N-glycoprofile together with indications of specificity of the observed changes for LF.

## Methods

### Reagents

All reagents were obtained from MilliporeSigma unless indicated otherwise. All enzymes were obtained from New England Biolabs (NEB), Ipswich, MA, USA.

### Rhesus macaque serum samples

The cohort of longitudinal serum samples from rhesus macaques has been described previously^83^. Briefly, serum was first collected from 7 healthy monkeys and 4 of the animals were then infected by subcutaneous infection with 130–180 infective third stage larvae of *B. malayi*. Serum was sampled again at 5, 12 and 15 wpi. All infected animals showed microfilariae in their blood by 12 wpi and impaired lymph flow when assessed at 16 wpi. Infection was confirmed by microscopic detection of microfilariae in blood. Details of disease progression and microfilariaemia for each animal are summarized in Supplementary Table S3 online.

### Ethical statement

Use of macaques and the experimental procedures performed in this study were reviewed and approved by The Institutional Animal Care and Use Committee (IACUC) at Bioqual Inc, Rockville, MA, USA and by the University of Illinois College of Medicine at Rockford, USA. Humane use of animals was performed according to the guidelines for the care and use of laboratory animals and with the rules formulated under the Animal Welfare Act by the U.S. Department of Agriculture in compliance with the Animal Research: Reporting of In Vivo Experiments (ARRIVE) guidelines. Ethical compliance, infection, treatment and animal maintenance have been reported in detail previously^83^.

### IgG purification from rhesus macaque serum

Aliquots of pre-infection serum from the 7 rhesus macaques were combined. IgG was purified from this serum pool using Protein G Spin Plates for IgG Screening (#45204, Thermo Scientific, Pierce Biotechnology, Rockford, IL). 5 µL of serum (processed in technical duplicates) was diluted with MilliQ water (MQ, 5 µL) then mixed with binding buffer (10 µL, 0.1 M NaHPO4 and 0.15 M NaCl, pH 7.2) and purified according to the manufacturer’s instructions. Purified IgG samples were concentrated and buffer exchanged to MQ using Vivaspin® centrifugal devices (5000 Da MWCO; #VS0111). Samples were transferred to microfuge tubes, dried using a speed-vacuum centrifuge (Speedvac) and resuspended in MQ for subsequent N-glycan analysis. Protein concentration was estimated by absorbance measurement using Nanodrop.

### MALDI-TOF-MS analysis

#### N-glycan release

A starting amount of 5 µL of the healthy rhesus macaque serum pool or 50 µg of purified serum IgG were mixed with PBS to reach a total volume of 100 µL. Samples were adjusted to 1.3% SDS and 0.1% β-mercaptoethanol. Denaturation was performed at 95 °C for 10 min. After cooling, Nonidet P-40 was added (1.3% final concentration). N-linked glycans were released from glycoproteins using Peptide-N-glycosidase (PNGase) F (17 mU) for 24 h at 37 °C. Released N-glycans were subsequently purified on octyldecylsilane (C18) cartridges (#7020-03 BAKERBOND® spe™, JT Baker®, Phillipsburg, NJ) equilibrated sequentially with acetronitrile (ACN), 60% ACN containing trifluoracetic acid (TFA, 0.1%) and MQ. Samples were mixed with 1 mL of MQ and applied to the C18 columns which were washed with 10% ACN followed by MQ. The column flow-throughs containing the carbohydrates were applied to a carbon column (Supelclean™ ENVI-Carb SPE) previously equilibrated sequentially with ACN, 50% ACN containing 0.1% TFA and MQ. Columns were washed with MQ and glycans were eluted with 25% ACN followed by 50% ACN containing 0.1% TFA. Collected N-glycan samples were split in two and dried down using a Speedvac. One half was redissolved in 50 µL MQ and directly labeled with anthranilic acid (2-AA), while the other half was subjected to a linkage-specific sialic acid derivatization prior to 2-AA labeling.

#### Ethylation and amidation

Linkage-specific derivatization of the sialic acids by ethyl esterification was essentially performed according to a previously developed protocol^53,54^. Briefly, the dried N-glycan eluates were first redissolved in 3 µL of MQ, then, 60 µL of freshly made ethylation reagent consisting of a mixture of N-(3-Dimethylaminopropyl)-N′-ethylcarbodiimide hydrochloride and hydroxybenzotriazole (both at 0.25 M) in ethanol were added. Ethyl esterification was performed for 30 min at 37 °C. 12 µL of 28% ammonium hydroxide was added to the reaction and incubation continued for a further 30 min at 37 °C. Samples were dried down and redissolved in 30 µL of MQ.

#### Anthranilic acid labeling

The derivatized glycans (ethylated, amidated) and the unmodified glycans from carbon column eluates both separately resuspended in MQ as described above were mixed with an equal volume of 2-AA labeling mix. 2-AA labeling mix was prepared by diluting 2-AA to a 48 mg/mL concentration and 2-picoline-borane complex to a 107 mg/mL concentration in DMSO:acetic acid (AcOH) (10:3). Labeling reactions were performed at 65° for 2 h.

#### Sample clean-up

Derivatized N-glycans were cleaned up using cotton HILIC solid phase extraction (SPE) following previously published methods^53,84^ with the following modifications. 20 µL pipette-tips packed with 3 mm cotton thread (180 μg, Pipoos, Utrecht, Netherlands) were washed with MQ and 85% ACN. Samples were loaded onto the tips by pipetting up and down 30 times before sequential washes with 85% ACN-0.1% TFA and 85% ACN. Glycans were eluted with 30 µL of MQ pipetted up and down 10 times through the cotton HILIC resin. For the remainder of the glycan samples (unmodified glycans), excess 2-AA labeling reagent was removed by SPE using Biogel P10 (#1504144 Bio-Rad, Hercules, CA). 200 µL of a 100 mg/mL solution of Biogel P10 in 10% ACN was loaded into the wells of a 96-well filter plate on top of a vacuum manifold device. The resins were washed sequentially with MQ and 80% ACN. Samples were brought to 75% ACN and loaded onto the resin. Following 4 washes with 80% ACN, samples were eluted from the Biogel P-10 twice with 200 µL of MQ. Samples were dried using a Speedvac and resolubilized in MQ. An additional clean-up step using C18 ZipTips (#ZTC18S096) was performed according to manufacturer’s instructions except for the final step, where the glycans were eluted in 50% ACN, 0.1% TFA mixed with 2,5-dihydroxybenzoic acid (DHB), at a concentration of 10 mg/ml (#8201346, Bruker Daltonics, Bremen, Germany). This facilitated direct sample loading to a 384-well steel polished target plate for MS analysis.

#### MALDI-TOF-MS and MALDI-TOF-MS/MS

2-AA labelled N-glycans were analyzed using MALDI-TOF-MS performed using an UltrafleXtreme® mass spectrometer (Bruker Daltonics) equipped with a 1 kHz Smartbeam II laser technology and controlled by the FlexControl 3.4 Build 119 software (Bruker Daltonics). Ethylated and amidated N-glycans, eluted in MQ during HILIC clean-up, were mixed in a 1:2 ratio with DHB matrix at a concentration of 20 mg/ml in 30% ACN and spotted onto a 384-well steel polished target plate for MALDI-TOF-MS analysis, while 2-AA labeled N-glycans processed through C18 ZipTips were directly spotted onto the target plate as described above. All spectra were recorded in the negative-ion reflectron mode using Bruker® peptide calibration mix (#8206195, Bruker Daltonics) for external calibration. Spectra were obtained over a mass window of m/z 700–4000 with ion suppression below m/z 700 for a minimum of 20,000 shots (2000 Hz) obtained by manual selection of “sweet spots”. The FlexAnalysis 3.4 Build 76 software was used for data processing including smoothing of the spectra (Savitzky Golay algorithm, peak width: m/z 0.06, 1 cycle), baseline subtraction (Tophat algorithm) and manual peak picking. Known non-glycan peaks such as glucose polymers were excluded. Possible compositions of deprotonated masses of the selected peaks were assigned using the GlycoPeakfinder® tool of the GlycoWorkBench software^85^ (http://www.eurocarbdb.org/applications/ms-tools, Version 3, June 2007) allowing the following residues within the search engine: 2–20 hexoses and N-acetylhexosamines, 0–5 deoxyhexoses (Fucoses), 0–4 Neu5Gc and 0–4 Neu5Ac. Linkage-specific derivatization of sialic acids resulted in mass differences of sialic acids compared to their unmodified variants of 291.10 Da for Neu5Ac and 307.09 Da for Neu5Gc. Derivatization of α2,6-linked sialic acids results in an increase of + 28.031 Da upon esterification^54^. Thus, we included the increment masses of 319.127 Da for α2,6-linked Neu5Ac and 335.307 Da for α2,6-linked Neu5Gc. In addition, amidation of α2,3-linked sialic acids yields side products showing a mass difference of − 0.984 Da corresponding to increment masses of 290.085 Da for α2,6-linked Neu5Ac and 306.249 Da for α2,6-linked Neu5Gc. The 2-AA label was also taken into account as a fixed reducing-end modification for composition assignment. Compositions were narrowed and confirmed by combining information derived from HILIC-UPLC analysis, glycosidase digestions (see below) and tandem MS (MS/MS). MS/MS was performed on underivatized, 2-AA labeled rhesus macaque IgG N-glycans. Selected ions were subjected to fragmentation analysis by MALDI-TOF/TOF using the UltrafleXtreme® mass spectrometer in negative-ion mode.

### HILIC-UPLC profiling of N-glycans

We used HILIC-UPLC to monitor the N-glycan profiles of rhesus macaques pre- and post-infection with *B. malayi* using the longitudinal set described above. We released serum N-glycans for each of the animals pre-infection (n = 7) to generate a baseline and for each of the animals subsequently infected with *B. malayi* (n = 4) at 5, 12 and 15 wpi. Each individual sample was processed separately, with technical duplicates to assess potential changes of the N-glycan profile upon infection. N-glycans in all samples were released and labeled with procainamide using the protocol optimized and described previously^32^. Briefly, N-glycan release was performed using Rapid™ PNGase F (#P0710) and directly followed by labeling. 12 µL of acidified procainamide (550 mg/mL procainamide stock solution in DMSO mixed with AcOH; 8:1 ratio) and 9 µL of sodium cyanoborohydride (200 mg/mL in MQ) were added to each deglycosylation reaction. Excess labeling reagent was then removed using HILIC clean-up plates (SNS-HIL, The Nest Group Inc., Southborough, MA, USA). The procainamide-labeled N-glycans were analyzed using HILIC-UPLC as described^32,51^. Briefly, a Waters Acquity H-class instrument (Waters Corp., Milford, MA, USA) composed of a binary solvent manager, a sample manager, a fluorescence detector (excitation wavelength 310 nm; detection wavelength 370 nm) and a QDa mass detector (settings: positive mode; target sampling rate: 10 point/sec; gain: 1; capillary voltage: 1.5 kV; probe temperature: 600 °C) was used for UPLC profiling. Glycans were separated using an Acquity BEH Amide Column (130 Å, 1.7 µm, 2.1 mm × 150 mm; Waters Corp.) with 50 mM ammonium formate, pH 4.4 as solvent A and acetonitrile as solvent B using a linear gradient of 70% to 53% solvent B at 0.56 ml/min for 25 min. Data from both the fluorescence and the mass detectors was acquired, processed and analyzed using Empower 3 software (Waters Corp.). Glucose units (GU) were assigned using a procainamide-labeled dextran ladder (#CPROC-GHP-30, Ludger, Abingdon, UK) and a fifth-order polynomial distribution curve.

### HILIC-UPLC data analysis and statistics

Peak areas of HILIC-UPLC spectra quantified using Empower 3 were exported and normalized to an arbitrary identical total peak area value of 10,000,000 for each spectrum. Statistical analysis was performed using the software R (version 4.0.0 Patched). The MSstats package^62^ (10.18129/B9.bioc.MSstats) was used to assess significant changes between time-points in the course of disease progression as described previously^51^. We performed this analysis on the longitudinal cohort consisting of the four animals described above. More specifically, for each animal, HILIC-UPLC profiles obtained at 5, 12 and 15 wpi were compared to their corresponding baseline profiles.

### Glycosidase digestions of released and labeled *N*-glycans

Released and labeled N-glycans from the healthy rhesus macaque serum pool and from rhesus macaque IgG were further characterized by enzymatic digestions. Sequential enzymatic digestions were performed using a panel of exoglycosidases. 8 µL of the serum N-glycan pool and 2 µL of the IgG N-glycans were digested with 1 µL of one or more exoglycosidase(s) to create a sequential digestion panel. For this purpose, α2–3,6,8,9 Neuraminidase A (#P0722), α1–2,4,6 Fucosidase O (#P0749), β1–4 Galactosidase S (#P0745) and β-N-Acetylglucosaminidase S (#P0744) were used. 1 µL of recommended buffer was added to each reaction and final reaction volumes were adjusted to 10 µL with MQ. Similarly, non-sequential digestions were performed by mixing 2 to 8 µL of the N-glycan samples with one of the following glycosidases in recommended amount and buffer: α2–3 Neuraminidase S (#P0743), α1–2,4,6 Fucosidase O (#P0749), α1–3,4,6 Galactosidase (#P0747), β1-4 Galactosidase S (#P0745) and β-N-Acetylglucosaminidase S (#P0744) or Endoglycosidase H (#P0702). Glycosidase sources and reaction conditions are further detailed in Supplementary Table S7 online. Undigested controls consisting of 2 to 8 µL of sample mixed with digestion reaction buffer and MQ were included for each experiment. Digestion reactions were incubated overnight at 37 °C prior to enzyme clean-up. MultiScreen® 96 well filter plates (#MAIPS4510) containing 0.45 µm hydrophobic high protein binding immobilon-P membrane was used as recommended for purification of the procainamide-labeled N-glycans while C18 Zip-tips were used as detailed above for purification of 2-AA-labeled N-glycans. Finally, digested N-glycan samples and undigested controls were analyzed using either HILIC-UPLC for procainamide-labeled N-glycans or MALDI-TOF-MS for 2-AA-labeled N-glycans as described above.

## Supplementary Information

Supplementary Information 1.

Supplementary Information 2.

Supplementary Information 3.

Supplementary Information 4.

Supplementary Information 5.

Supplementary Information 6.

Supplementary Information 7.

Supplementary Information 8.

## Data Availability

HILIC-UPLC and MALDI-TOF-MS(/MS) spectra supporting our findings have been made available in the supplementary data. In addition, all raw MALDI-TOF-MS and MALDI-TOF-MS/MS data presented in this study (both from main and supplementary figures) have been deposited in Glycopost (https://glycopost.glycosmos.org/, project ID: GPST000274).

## Abbreviations

2-AA: Anthranilic acid
ACN: Acetronitrile
AcOH: Acetic acid
*B. malayi*: *Brugia malayi*
*B. timori*: *Brugia timori*
C18: Octyldecylsilane
CMAH: CMP-N-acetylneuraminic acid hydroxylase
*D. immitis*: *Dirofilaria immitis*
DHB: 2,5-Dihydroxybenzoic acid
DMSO: Dimethyl sulfoxide
EDC: N-(3-Dimethylaminopropyl)-N′-ethylcarbodiimide hydrochloride
FcγRs: Fc γ receptors
GlcNAc: N-acetylglucosamine
GU: Glucose units
HILIC: Hydrophilic interaction chromatography
Ig: Immunoglobulin
LF: Lymphatic filariasis
MALDI-TOF-MS: Matrix-assisted laser desorption/ionization time of flight mass spectrometry
MQ: MilliQ water
MS: Mass spectrometry
MS/MS: Tandem MS
NEB: New England Biolabs
Neu5Ac: *N-*Acetylneuraminic acid
Neu5Gc: *N-*Glycolylneuraminic acid
PBS: Phosphate-buffered saline
PNGase F: Peptide-N-glycosidase F
SDS: Sodium dodecyl sulfate
SPE: Solid phase extraction
TFA: Trifluoracetic acid
UPLC: Ultraperformance liquid chromatography
*W. bancrofti*: *Wuchereria bancrofti*
wpi: Weeks post-infection
