# Supplementary material for: Alteration of rhesus macaque serum N-glycome during infection with the human parasitic filarial nematode Brugia malayi
Source: Sci Rep. 2022 Sep 21;12:15763. doi: 10.1038/s41598-022-19964-1 (PMC9491660; doi:10.1038/s41598-022-19964-1)
Supplement: Supplementary file 1 — Supplementary Information 1. [file 41598_2022_19964_MOESM1_ESM.pptx]

## Slide 1
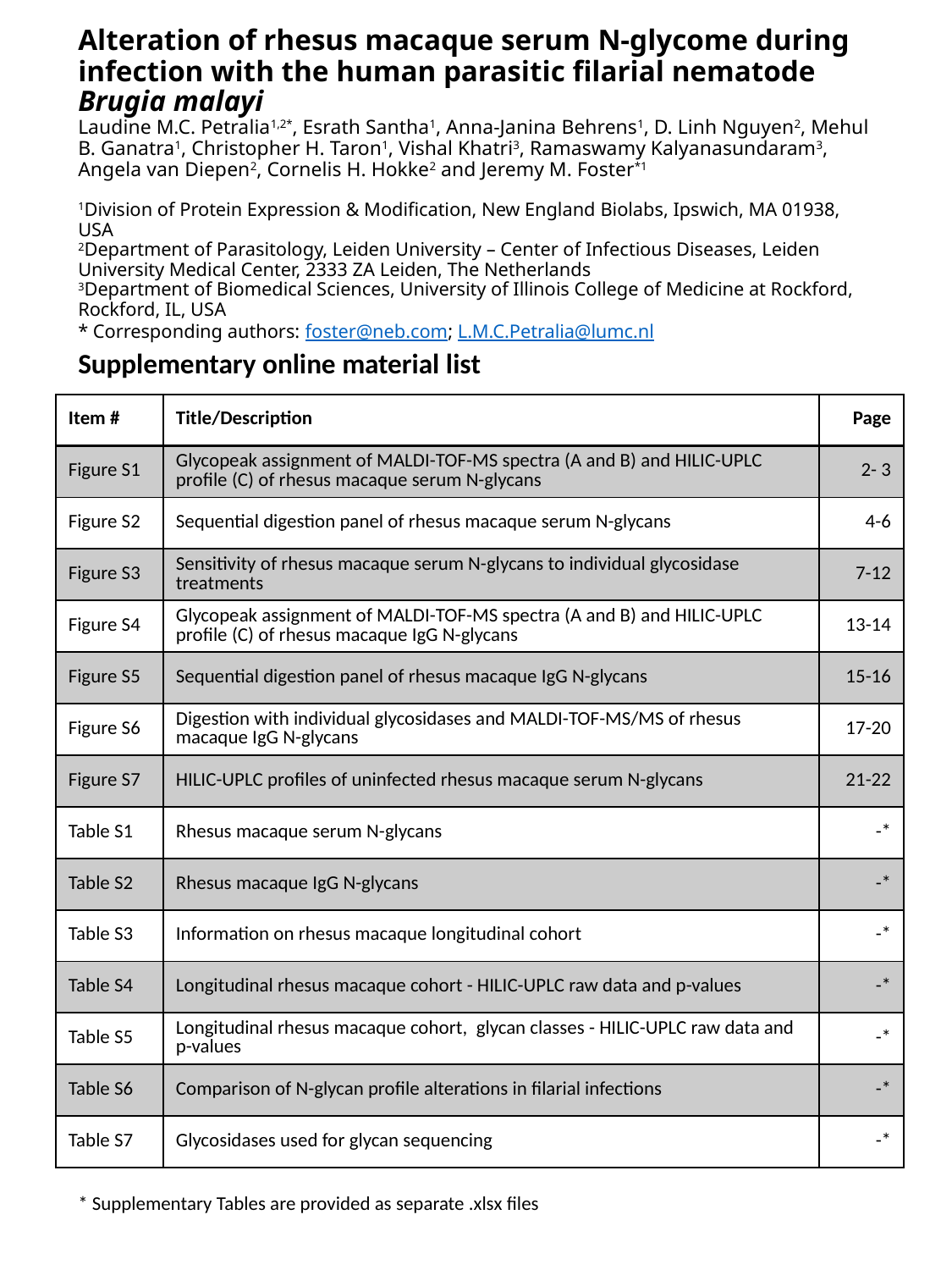

# Alteration of rhesus macaque serum N-glycome during infection with the human parasitic filarial nematode Brugia malayi Laudine M.C. Petralia1,2*, Esrath Santha1, Anna-Janina Behrens1, D. Linh Nguyen2, Mehul B. Ganatra1, Christopher H. Taron1, Vishal Khatri3, Ramaswamy Kalyanasundaram3, Angela van Diepen2, Cornelis H. Hokke2 and Jeremy M. Foster*1 1Division of Protein Expression & Modification, New England Biolabs, Ipswich, MA 01938, USA 2Department of Parasitology, Leiden University – Center of Infectious Diseases, Leiden University Medical Center, 2333 ZA Leiden, The Netherlands 3Department of Biomedical Sciences, University of Illinois College of Medicine at Rockford, Rockford, IL, USA * Corresponding authors: foster@neb.com; L.M.C.Petralia@lumc.nl
Supplementary online material list
* Supplementary Tables are provided as separate .xlsx files
| Item # | Title/Description | Page |
| --- | --- | --- |
| Figure S1 | Glycopeak assignment of MALDI-TOF-MS spectra (A and B) and HILIC-UPLC profile (C) of rhesus macaque serum N-glycans | 2- 3 |
| Figure S2 | Sequential digestion panel of rhesus macaque serum N-glycans | 4-6 |
| Figure S3 | Sensitivity of rhesus macaque serum N-glycans to individual glycosidase treatments | 7-12 |
| Figure S4 | Glycopeak assignment of MALDI-TOF-MS spectra (A and B) and HILIC-UPLC profile (C) of rhesus macaque IgG N-glycans | 13-14 |
| Figure S5 | Sequential digestion panel of rhesus macaque IgG N-glycans | 15-16 |
| Figure S6 | Digestion with individual glycosidases and MALDI-TOF-MS/MS of rhesus macaque IgG N-glycans | 17-20 |
| Figure S7 | HILIC-UPLC profiles of uninfected rhesus macaque serum N-glycans | 21-22 |
| Table S1 | Rhesus macaque serum N-glycans | -\* |
| Table S2 | Rhesus macaque IgG N-glycans | -\* |
| Table S3 | Information on rhesus macaque longitudinal cohort | -\* |
| Table S4 | Longitudinal rhesus macaque cohort - HILIC-UPLC raw data and p-values | -\* |
| Table S5 | Longitudinal rhesus macaque cohort, glycan classes - HILIC-UPLC raw data and p-values | -\* |
| Table S6 | Comparison of N-glycan profile alterations in filarial infections | -\* |
| Table S7 | Glycosidases used for glycan sequencing | -\* |

## Slide 2
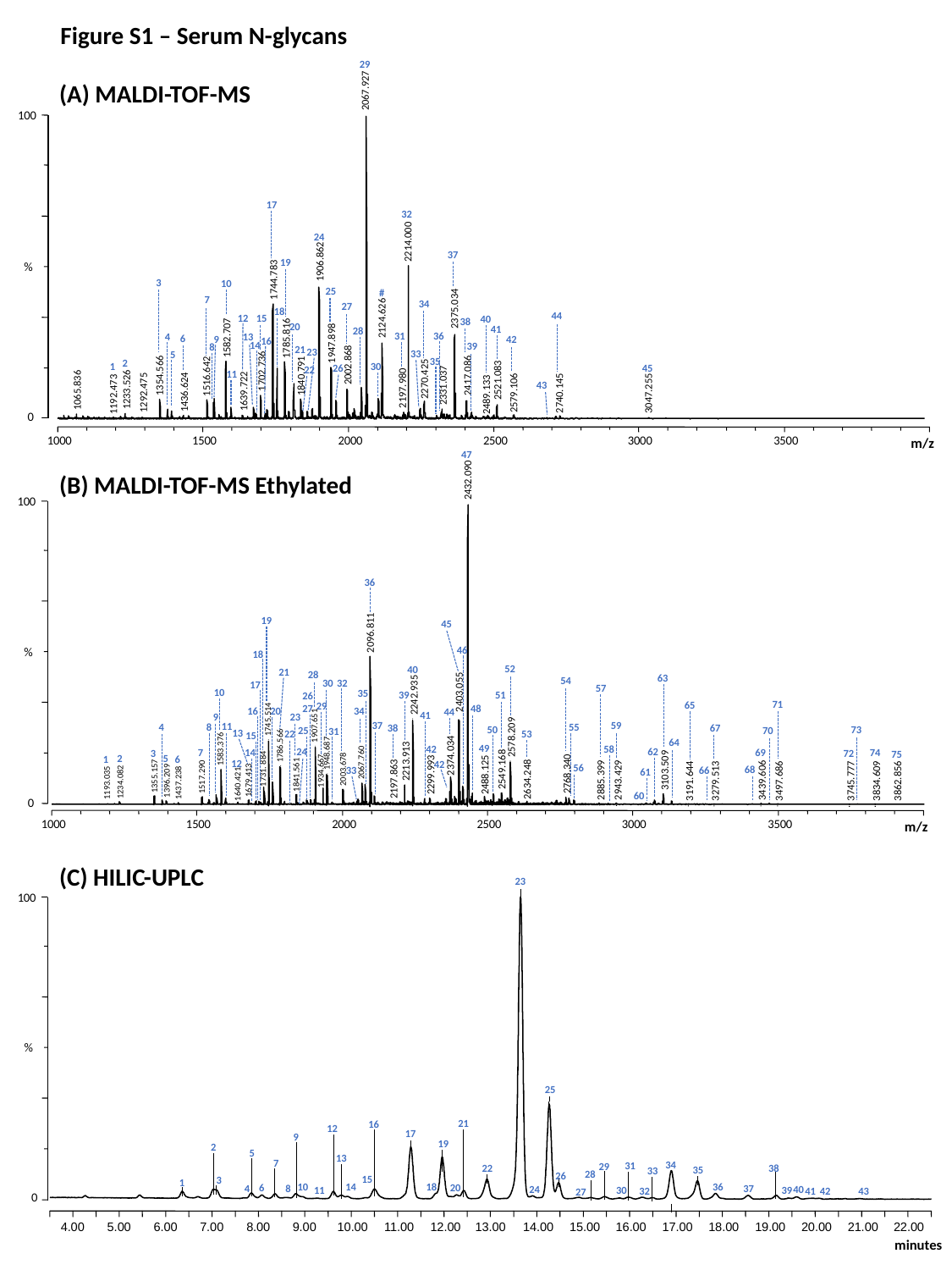

Figure S1 – Serum N-glycans
29
(A) MALDI-TOF-MS
2067.927
100
17
32
24
2214.000
37
19
1906.862
%
3
10
1744.783
25
#
7
34
27
18
2375.034
44
12
15
40
38
2124.626
20
41
28
31
36
4
13
6
42
9
16
1582.707
1785.816
14
39
8
1947.898
21
23
33
5
35
2
30
1
26
45
22
2002.868
11
1702.736
1354.566
2417.086
1840.791
1516.642
43
2270.425
2521.083
2331.037
2197.980
1233.526
1065.836
1639.722
1436.624
1292.475
2579.106
2740.145
3047.255
1192.473
2489.133
0
2500
1000
1500
2000
3000
3500
m/z
47
(B) MALDI-TOF-MS Ethylated
2432.090
100
36
19
45
2096.811
46
18
%
52
40
21
28
63
54
32
30
17
57
10
35
39
51
26
27
2403.055
2242.935
71
65
29
48
20
34
16
44
41
9
23
37
59
11
1745.514
55
8
4
38
67
50
73
25
70
31
1907.651
13
53
22
15
64
2578.209
49
42
58
24
62
14
69
7
74
72
3
1786.566
75
1583.376
5
2
6
1
1948.687
2374.034
12
42
2213.913
56
68
66
33
2067.760
61
2549.168
1731. 884
3103.509
2003.678
1934.667
2768.340
2488.125
2299.993
1841.561
1355.157
1517.290
2197.863
2634.248
2885.399
2943.429
3439.606
3191.644
3279.513
3497.686
3745.777
3834.609
3862.856
1679.413
1396.209
1234.082
1193.035
1437.238
1640.421
60
0
1000
1500
2000
2500
3000
3500
m/z
(C) HILIC-UPLC
23
100
%
25
21
16
12
17
9
19
2
5
13
7
34
31
29
38
22
35
33
28
26
15
3
1
14
18
36
10
20
6
8
37
4
24
40
11
30
39
32
43
41
42
27
0
22.00
4.00
5.00
6.00
7.00
8.00
9.00
10.00
11.00
12.00
13.00
14.00
15.00
16.00
17.00
18.00
19.00
20.00
21.00
minutes

## Slide 3
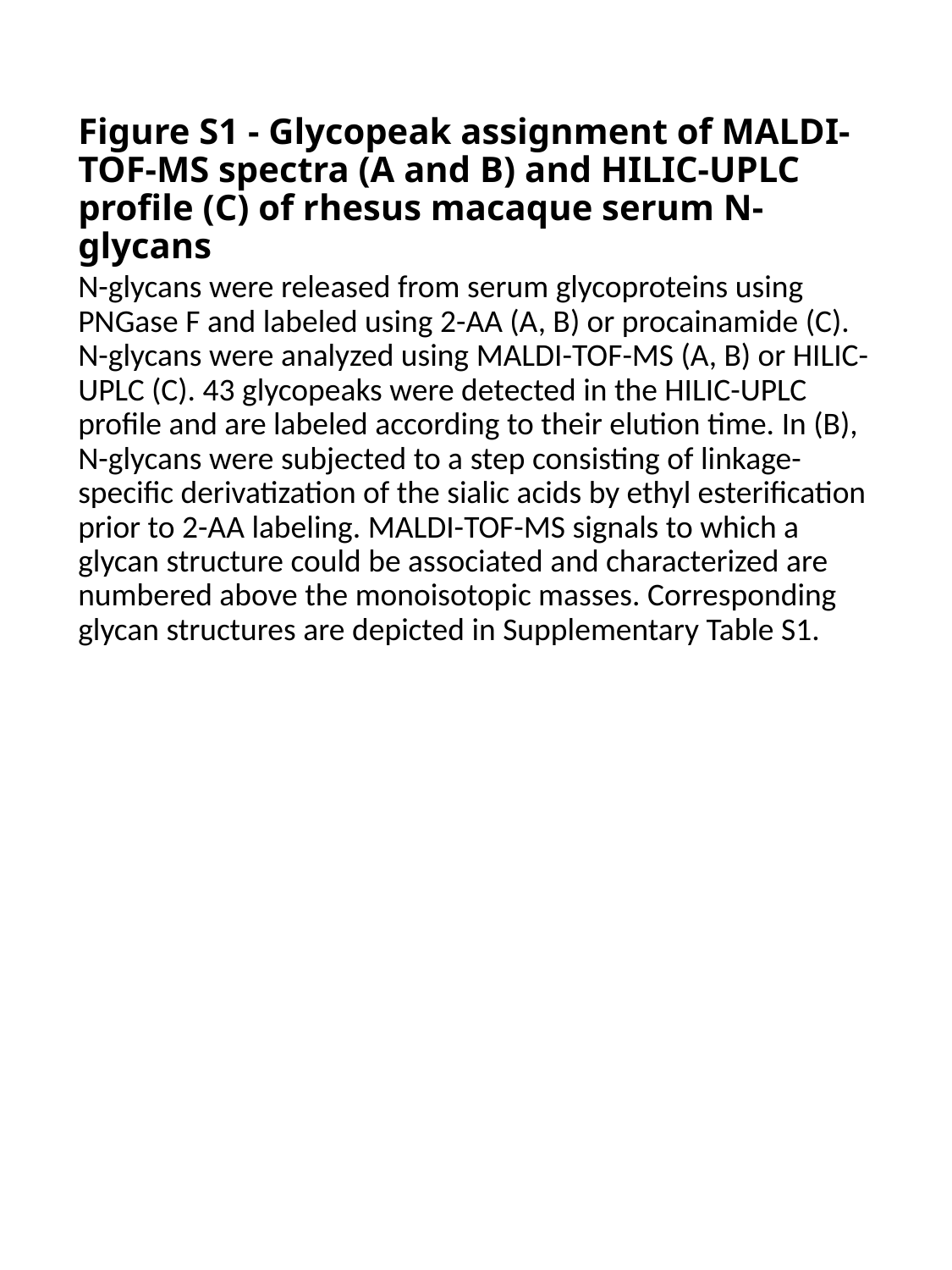

# Figure S1 - Glycopeak assignment of MALDI-TOF-MS spectra (A and B) and HILIC-UPLC profile (C) of rhesus macaque serum N-glycans
N-glycans were released from serum glycoproteins using PNGase F and labeled using 2-AA (A, B) or procainamide (C). N-glycans were analyzed using MALDI-TOF-MS (A, B) or HILIC-UPLC (C). 43 glycopeaks were detected in the HILIC-UPLC profile and are labeled according to their elution time. In (B), N-glycans were subjected to a step consisting of linkage-specific derivatization of the sialic acids by ethyl esterification prior to 2-AA labeling. MALDI-TOF-MS signals to which a glycan structure could be associated and characterized are numbered above the monoisotopic masses. Corresponding glycan structures are depicted in Supplementary Table S1.

## Slide 4
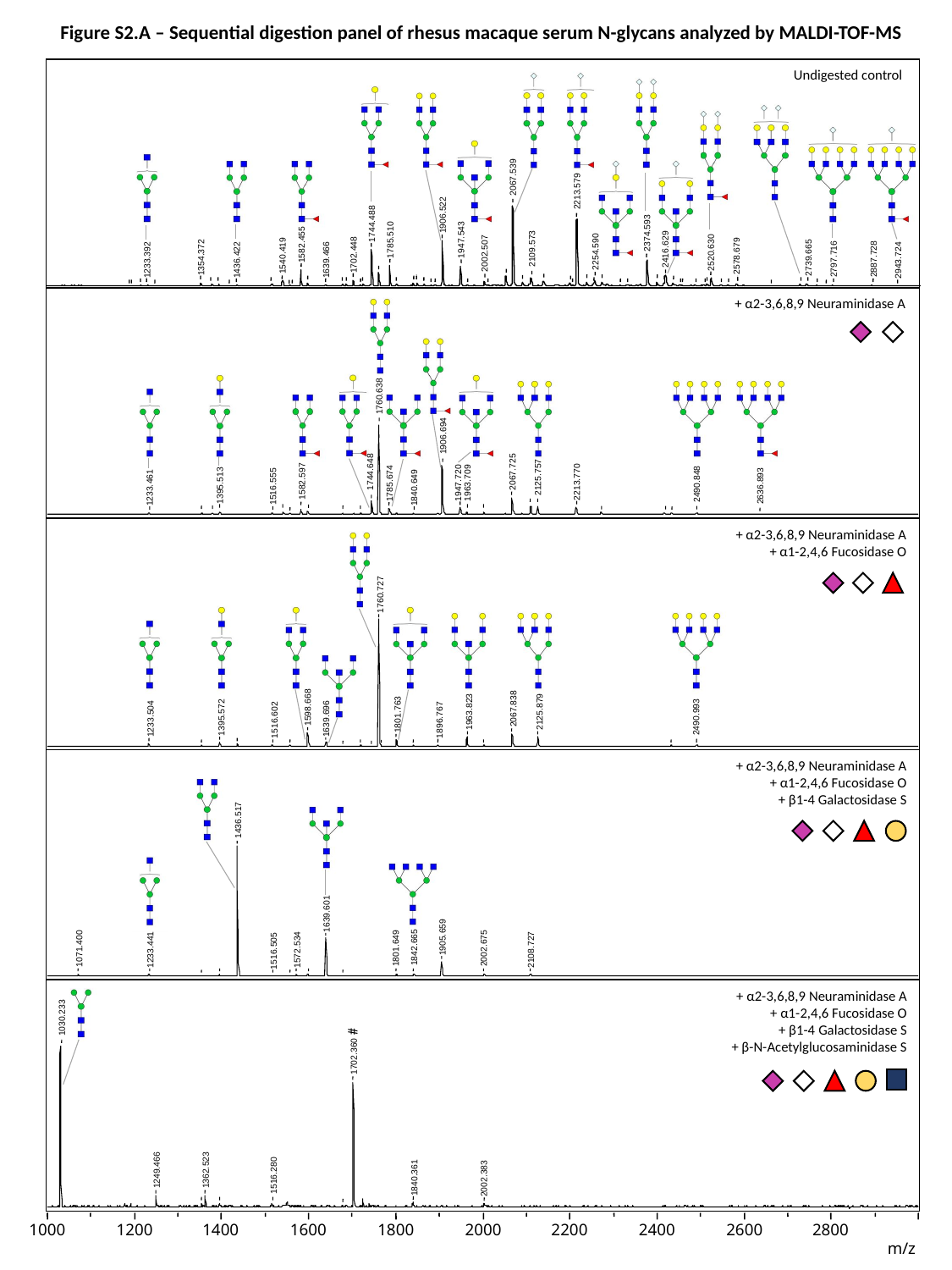

Figure S2.A – Sequential digestion panel of rhesus macaque serum N-glycans analyzed by MALDI-TOF-MS
Undigested control
2067.539
2213.579
1906.522
1744.488
2374.593
1947.543
1785.510
1582.455
2109.573
2416.629
2254.590
2520.630
2002.507
1702.448
1540.419
2578.679
1354.372
2739.665
2797.716
2887.728
1233.392
1436.422
1639.466
2943.724
+ α2-3,6,8,9 Neuraminidase A
1760.638
1906.694
1744.648
2067.725
2125.757
1582.597
2213.770
1947.720
1963.709
1785.674
2490.848
1395.513
2636.893
1516.555
1233.461
1840.649
+ α2-3,6,8,9 Neuraminidase A
+ α1-2,4,6 Fucosidase O
1760.727
1598.668
2067.838
1963.823
2125.879
1801.763
2490.993
1395.572
1639.696
1233.504
1516.602
1896.767
+ α2-3,6,8,9 Neuraminidase A
+ α1-2,4,6 Fucosidase O
+ β1-4 Galactosidase S
1436.517
1639.601
1905.659
1842.665
1801.649
2002.675
1071.400
1233.441
1572.534
2108.727
1516.505
+ α2-3,6,8,9 Neuraminidase A
+ α1-2,4,6 Fucosidase O
+ β1-4 Galactosidase S
+ β-N-Acetylglucosaminidase S
#
1030.233
1702.360
1249.466
1362.523
1516.280
1840.361
2002.383
1000
1200
1400
1600
1800
2000
2200
2400
2600
2800
m/z

## Slide 5
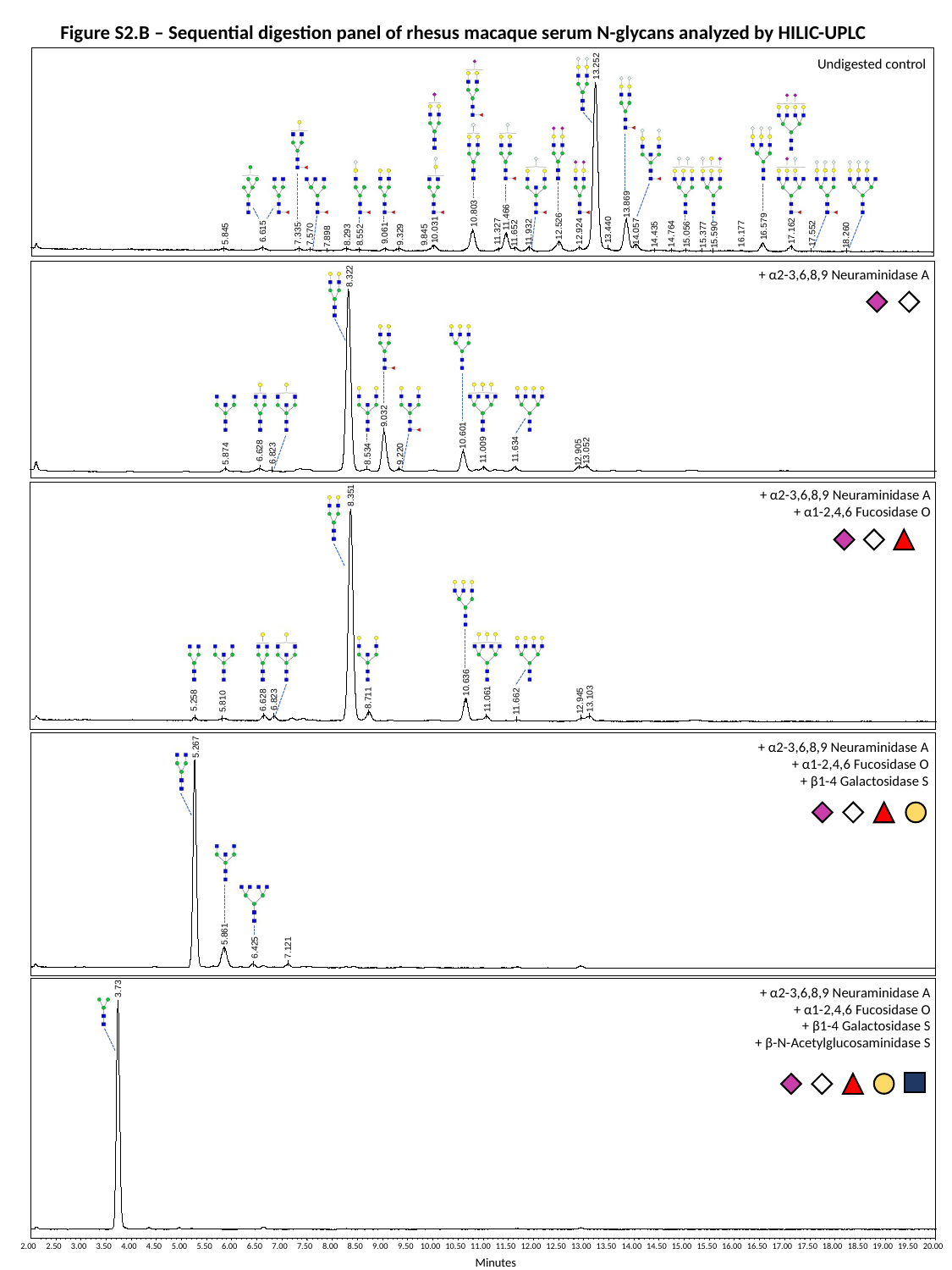

Figure S2.B – Sequential digestion panel of rhesus macaque serum N-glycans analyzed by HILIC-UPLC
Undigested control
13.252
13.869
10.803
11.466
12.526
16.579
10.031
13.440
17.162
12.924
11.327
14.057
6.615
11.932
11.652
17.552
14.764
7.335
5.845
16.177
7.570
14.435
15.056
9.329
15.377
 9.061
8.293
15.590
9.845
8.552
18.260
7.898
+ α2-3,6,8,9 Neuraminidase A
8.322
9.032
10.601
11.634
11.009
6.628
13.052
12.905
6.823
5.874
9.220
8.534
+ α2-3,6,8,9 Neuraminidase A
+ α1-2,4,6 Fucosidase O
 8.351
10.636
8.711
13.103
11.061
6.823
5.258
12.945
 6.628
11.662
5.810
+ α2-3,6,8,9 Neuraminidase A
+ α1-2,4,6 Fucosidase O
+ β1-4 Galactosidase S
 5.267
5.861
7.121
 6.425
+ α2-3,6,8,9 Neuraminidase A
+ α1-2,4,6 Fucosidase O
+ β1-4 Galactosidase S
+ β-N-Acetylglucosaminidase S
 3.73
2.00
2.50
3.00
3.50
4.00
4.50
5.00
5.50
6.00
6.50
7.00
7.50
8.00
8.50
9.00
9.50
10.00
10.50
11.00
11.50
12.00
12.50
13.00
13.50
14.00
14.50
15.00
15.50
16.00
16.50
17.00
17.50
18.00
18.50
19.00
19.50
20.00
Minutes

## Slide 6
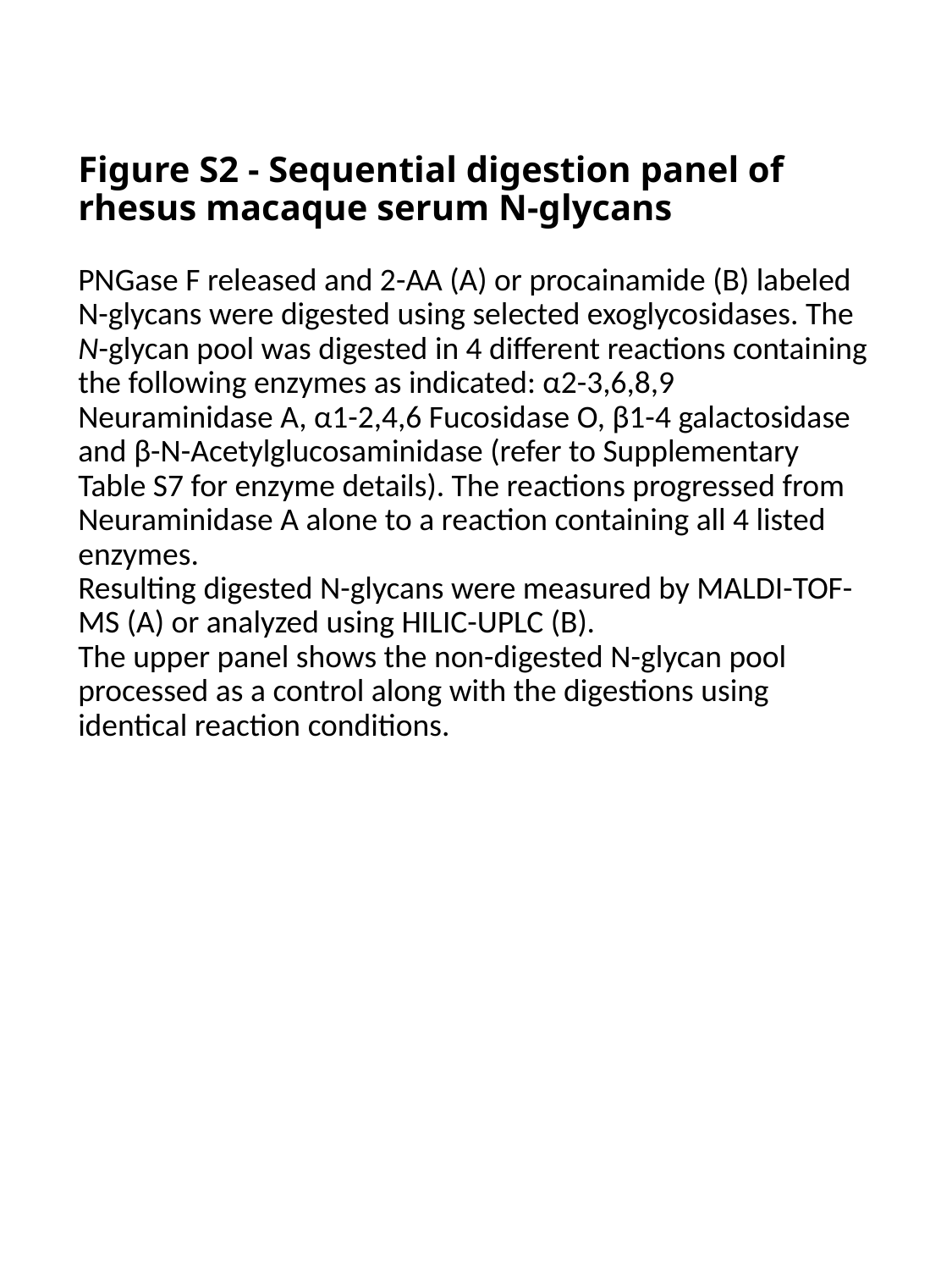

# Figure S2 - Sequential digestion panel of rhesus macaque serum N-glycans
PNGase F released and 2-AA (A) or procainamide (B) labeled N-glycans were digested using selected exoglycosidases. The N-glycan pool was digested in 4 different reactions containing the following enzymes as indicated: α2-3,6,8,9 Neuraminidase A, α1-2,4,6 Fucosidase O, β1-4 galactosidase and β-N-Acetylglucosaminidase (refer to Supplementary Table S7 for enzyme details). The reactions progressed from Neuraminidase A alone to a reaction containing all 4 listed enzymes.
Resulting digested N-glycans were measured by MALDI-TOF-MS (A) or analyzed using HILIC-UPLC (B).
The upper panel shows the non-digested N-glycan pool processed as a control along with the digestions using identical reaction conditions.

## Slide 7
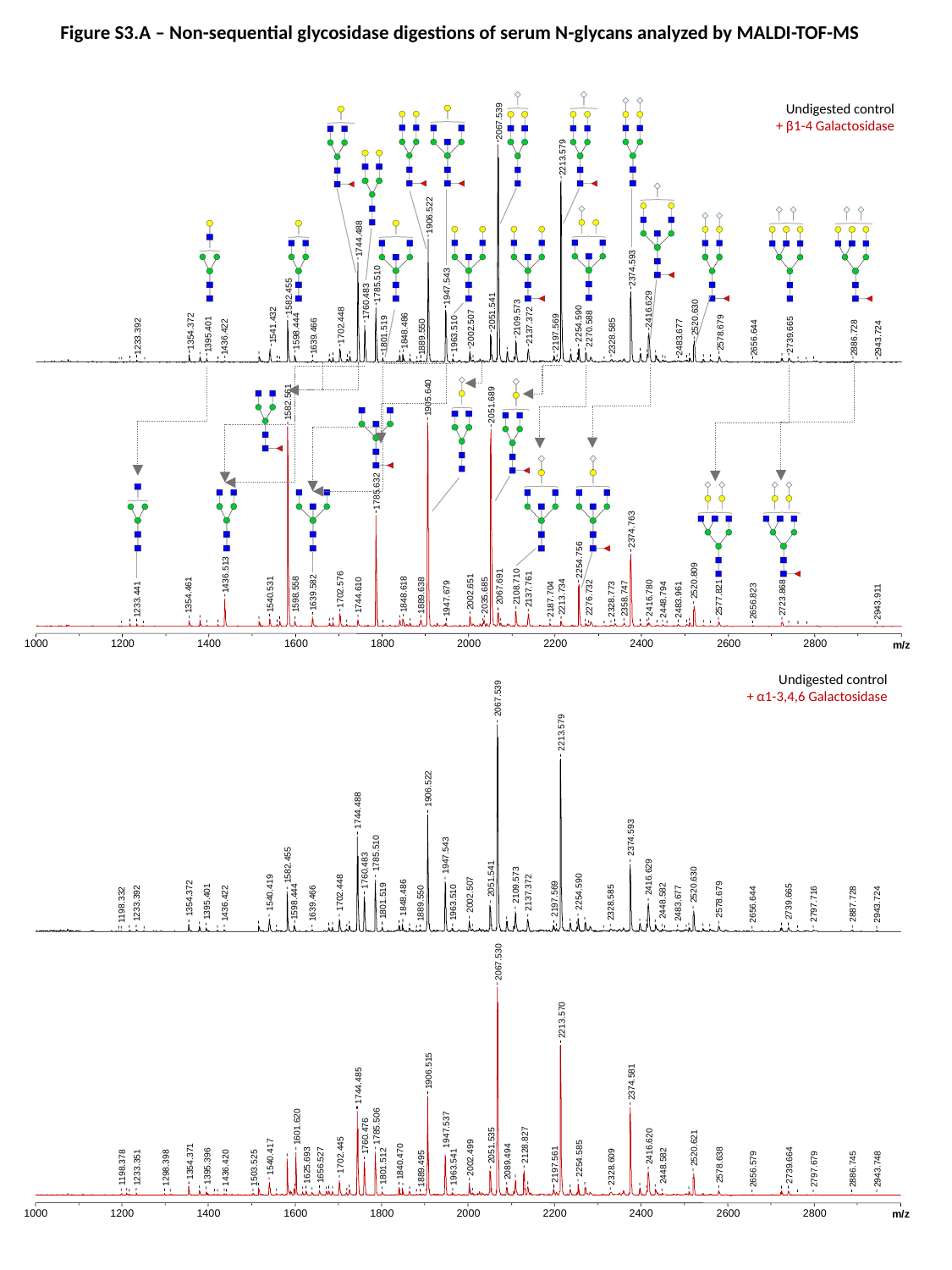

Figure S3.A – Non-sequential glycosidase digestions of serum N-glycans analyzed by MALDI-TOF-MS
Undigested control
+ β1-4 Galactosidase
2067.539
2213.579
1906.522
1744.488
2374.593
1785.510
1947.543
1582.455
1760.483
2416.629
2051.541
2109.573
2520.630
2254.590
1541.432
1702.448
2137.372
2002.507
2270.588
1848.486
1354.372
1598.444
2197.569
2578.679
1801.519
1963.510
1395.401
2739.665
2328.585
1639.466
1233.392
1436.422
1889.550
2483.677
2656.644
2886.728
2943.724
1905.640
1582.561
2051.689
1785.632
2374.763
2254.756
1436.513
2520.809
2067.691
2108.710
2137.761
1702.576
2002.651
1639.582
1848.618
1540.531
1598.558
1354.461
1744.610
1889.638
2035.685
2213.734
2276.732
2577.821
2723.868
2416.780
1947.679
2358.747
2187.704
2328.773
1233.441
2448.794
2483.961
2656.823
2943.911
1000
1200
1400
1600
1800
2000
2200
2400
2600
2800
m/z
Undigested control
+ α1-3,4,6 Galactosidase
2067.539
2213.579
1906.522
1744.488
2374.593
1785.510
1947.543
1582.455
1760.483
2416.629
2051.541
2520.630
2109.573
2254.590
1540.419
1702.448
2137.372
2002.507
1848.486
1354.372
2197.569
2578.679
1801.519
2448.582
1598.444
1395.401
2739.665
1963.510
2328.585
1233.392
1436.422
1639.466
1889.550
2483.677
2797.716
2887.728
1198.332
2656.644
2943.724
2067.530
2213.570
1906.515
2374.581
1744.485
1785.506
1601.620
1947.537
1760.476
2128.827
2051.535
2416.620
2520.621
1702.445
1540.417
2002.499
2254.585
1354.371
1840.470
2089.494
2197.561
2578.638
1656.527
2739.664
1625.693
2448.582
1395.396
1801.512
1963.541
2328.609
1198.378
1233.351
1298.398
1436.420
1503.525
1889.495
2656.579
2797.679
2886.745
2943.748
1000
1200
1400
1600
1800
2000
2200
2400
2600
2800
m/z

## Slide 8
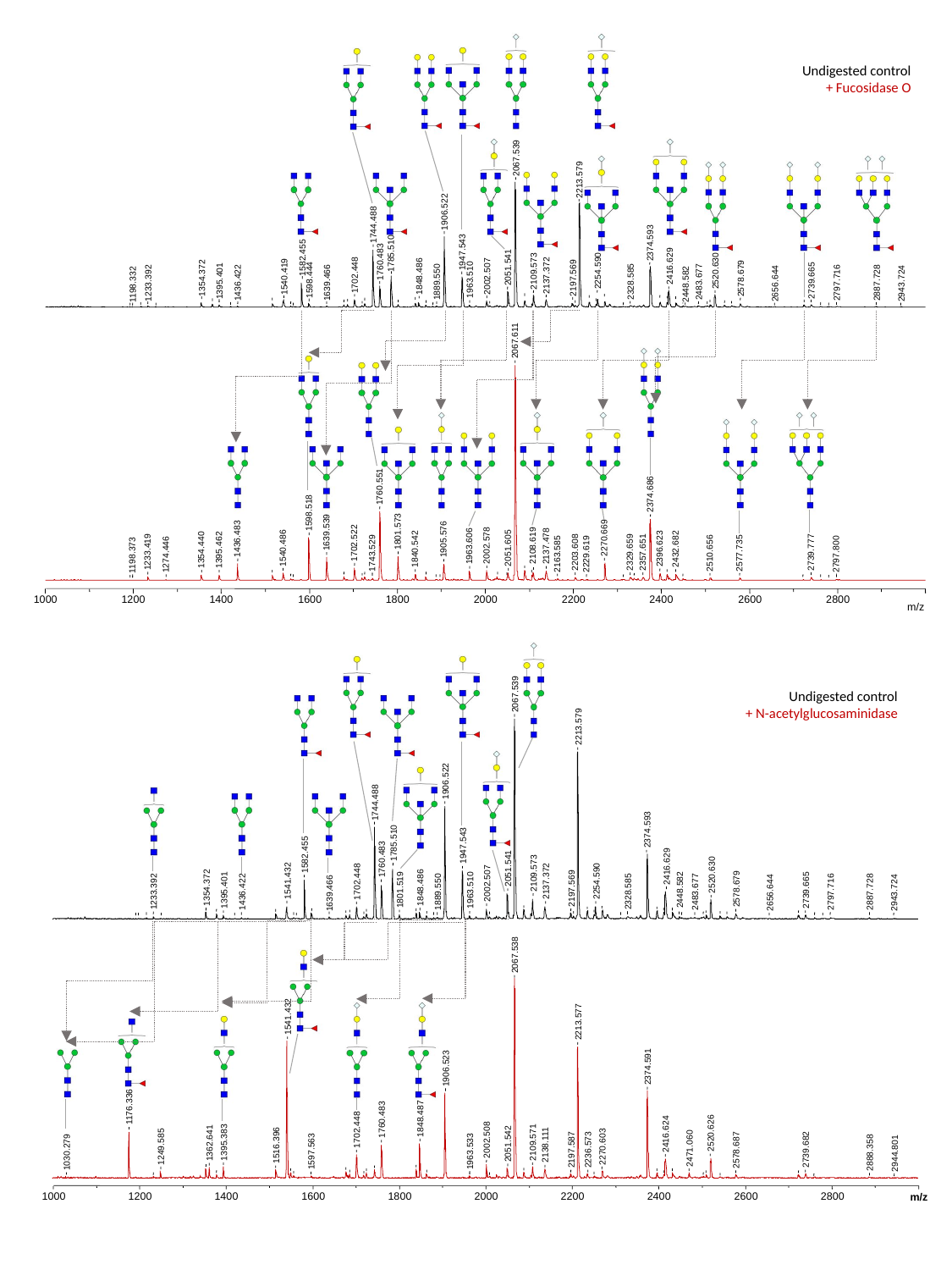

Undigested control
+ Fucosidase O
2067.539
2213.579
1906.522
1744.488
2374.593
1947.543
1785.510
1582.455
1760.483
2416.629
2051.541
2254.590
2520.630
2109.573
1702.448
2137.372
1848.486
2002.507
1540.419
1354.372
2197.569
2578.679
1963.510
1598.444
2739.665
1395.401
2328.585
1889.550
2483.677
2797.716
2887.728
1436.422
1639.466
1233.392
2656.644
2943.724
2448.582
1198.332
2067.611
1760.551
2374.686
1598.518
1801.573
1639.539
2270.669
1436.483
1905.576
1702.522
2002.578
2108.619
1963.606
2137.478
2051.605
1540.486
2396.623
1840.542
2432.682
1354.440
1395.462
1233.419
2203.608
2329.659
2357.651
2739.777
1743.529
2510.656
2577.735
2163.585
2229.619
2797.800
1274.446
1198.373
1000
1200
1400
1600
1800
2000
2200
2400
2600
2800
m/z
Undigested control
+ N-acetylglucosaminidase
2067.539
2213.579
1906.522
1744.488
2374.593
1785.510
1947.543
1582.455
1760.483
2416.629
2051.541
2109.573
2520.630
1541.432
2137.372
2254.590
1702.448
2002.507
1354.372
1848.486
2197.569
2578.679
1801.519
2448.582
1395.401
1963.510
2739.665
1233.392
2328.585
1436.422
1889.550
2483.677
2797.716
2887.728
2656.644
2943.724
1639.466
2067.538
1541.432
2213.577
2374.591
1906.523
1176.336
1848.487
1760.483
1702.448
2520.626
2416.624
2002.508
1395.383
2109.571
1362.641
2051.542
2138.111
1516.396
1249.585
2270.603
2471.060
2739.682
2197.587
2236.573
2578.687
1597.563
1963.533
1030.279
2888.358
2944.801
1000
1200
1400
1600
1800
2000
2200
2400
2600
2800
m/z

## Slide 9
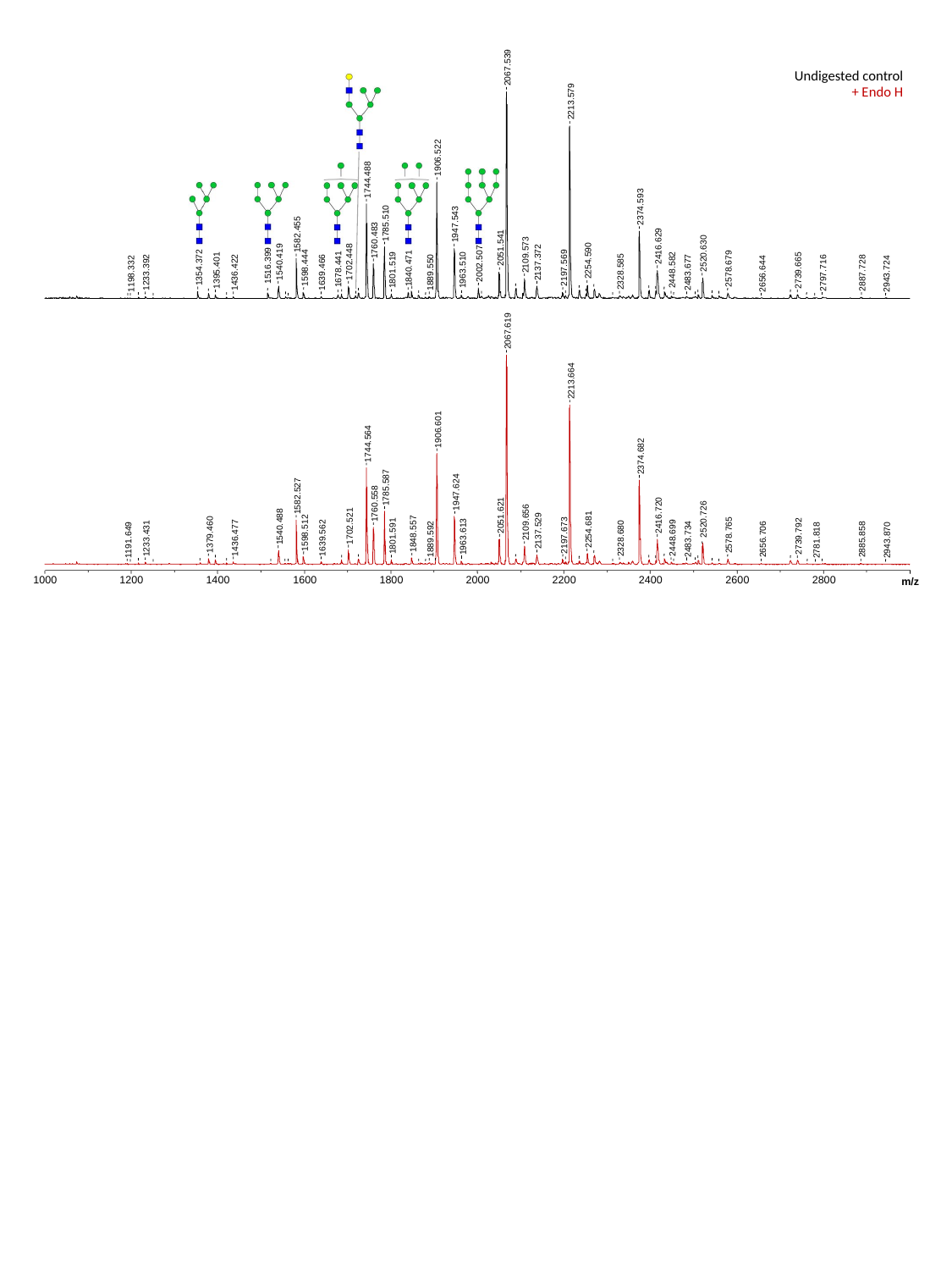

Undigested control
+ Endo H
2067.539
2213.579
1906.522
1744.488
2374.593
1785.510
1947.543
1582.455
1760.483
2416.629
2051.541
2520.630
2109.573
2254.590
1540.419
1702.448
2137.372
2002.507
1516.399
1354.372
1598.444
2197.569
1840.471
2578.679
1678.441
1801.519
1963.510
2448.582
1395.401
2739.665
2328.585
1233.392
1436.422
1639.466
1889.550
2483.677
2797.716
2887.728
1198.332
2656.644
2943.724
2067.619
2213.664
1906.601
1744.564
2374.682
1785.587
1947.624
1582.527
1760.558
2051.621
2416.720
2520.726
2109.656
1702.521
1540.488
2254.681
2137.529
1598.512
1848.557
1379.460
2578.765
2197.673
1801.591
2739.792
1963.613
1436.477
1639.562
2448.699
1233.431
2328.680
1889.592
2483.734
2656.706
2885.858
1191.649
2781.818
2943.870
1000
1200
1400
1600
1800
2000
2200
2400
2600
2800
m/z

## Slide 10
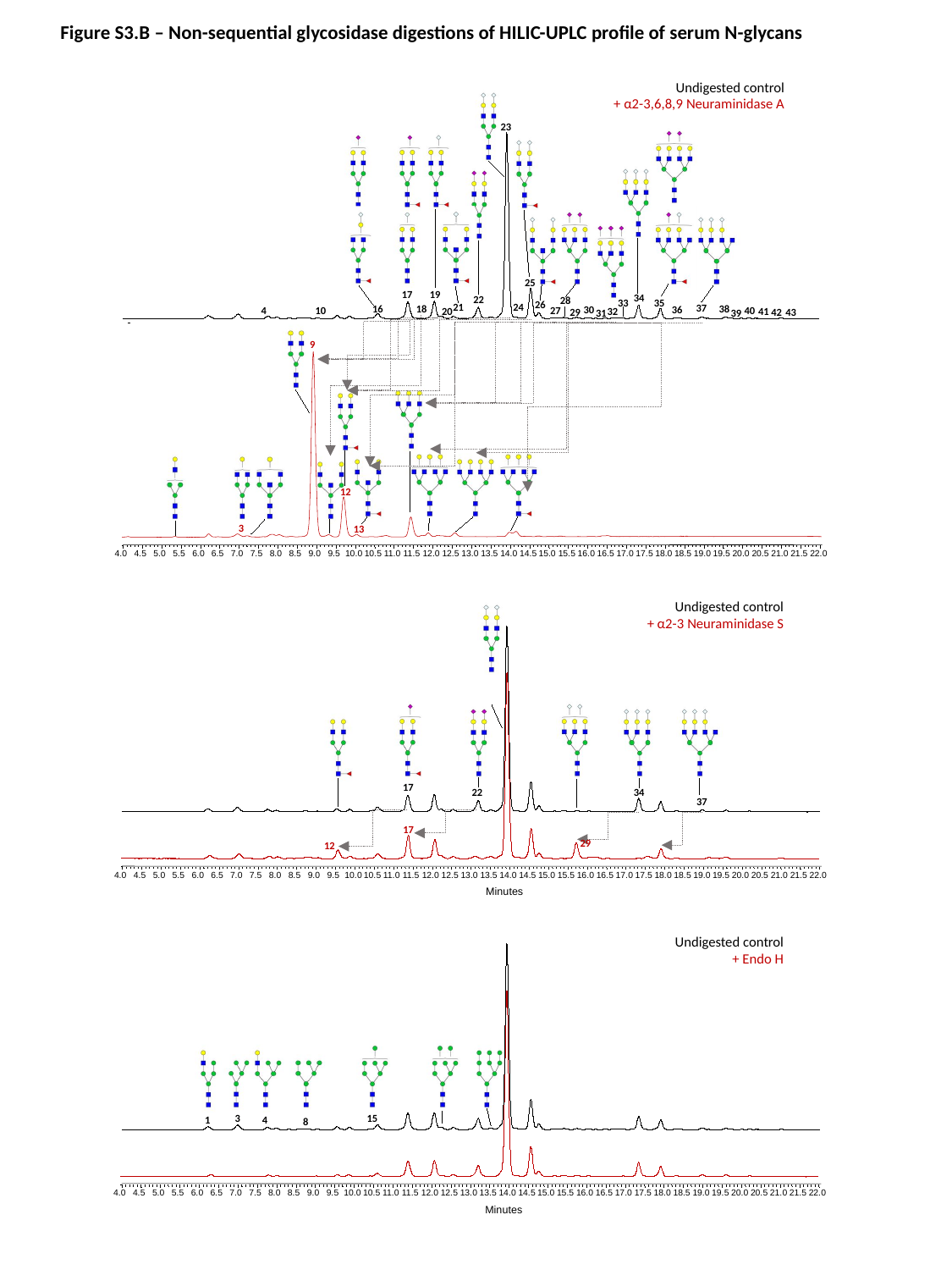

Figure S3.B – Non-sequential glycosidase digestions of HILIC-UPLC profile of serum N-glycans
Undigested control
+ α2-3,6,8,9 Neuraminidase A
23
25
19
17
34
22
28
33
35
26
24
21
37
16
18
38
36
30
10
4
27
40
32
41
20
29
42
43
31
39
 9
 12
 3
 13
4.0
4.5
5.0
5.5
6.0
6.5
7.0
7.5
8.0
8.5
9.0
9.5
10.0
10.5
11.0
11.5
12.0
12.5
13.0
13.5
14.0
14.5
15.0
15.5
16.0
16.5
17.0
17.5
18.0
18.5
19.0
19.5
20.0
20.5
21.0
21.5
22.0
Undigested control
+ α2-3 Neuraminidase S
17
34
22
37
 17
29
 12
4.0
4.5
5.0
5.5
6.0
6.5
7.0
7.5
8.0
8.5
9.0
9.5
10.0
10.5
11.0
11.5
12.0
12.5
13.0
13.5
14.0
14.5
15.0
15.5
16.0
16.5
17.0
17.5
18.0
18.5
19.0
19.5
20.0
20.5
21.0
21.5
22.0
Minutes
Undigested control
+ Endo H
3
15
1
4
8
4.0
4.5
5.0
5.5
6.0
6.5
7.0
7.5
8.0
8.5
9.0
9.5
10.0
10.5
11.0
11.5
12.0
12.5
13.0
13.5
14.0
14.5
15.0
15.5
16.0
16.5
17.0
17.5
18.0
18.5
19.0
19.5
20.0
20.5
21.0
21.5
22.0
Minutes

## Slide 11
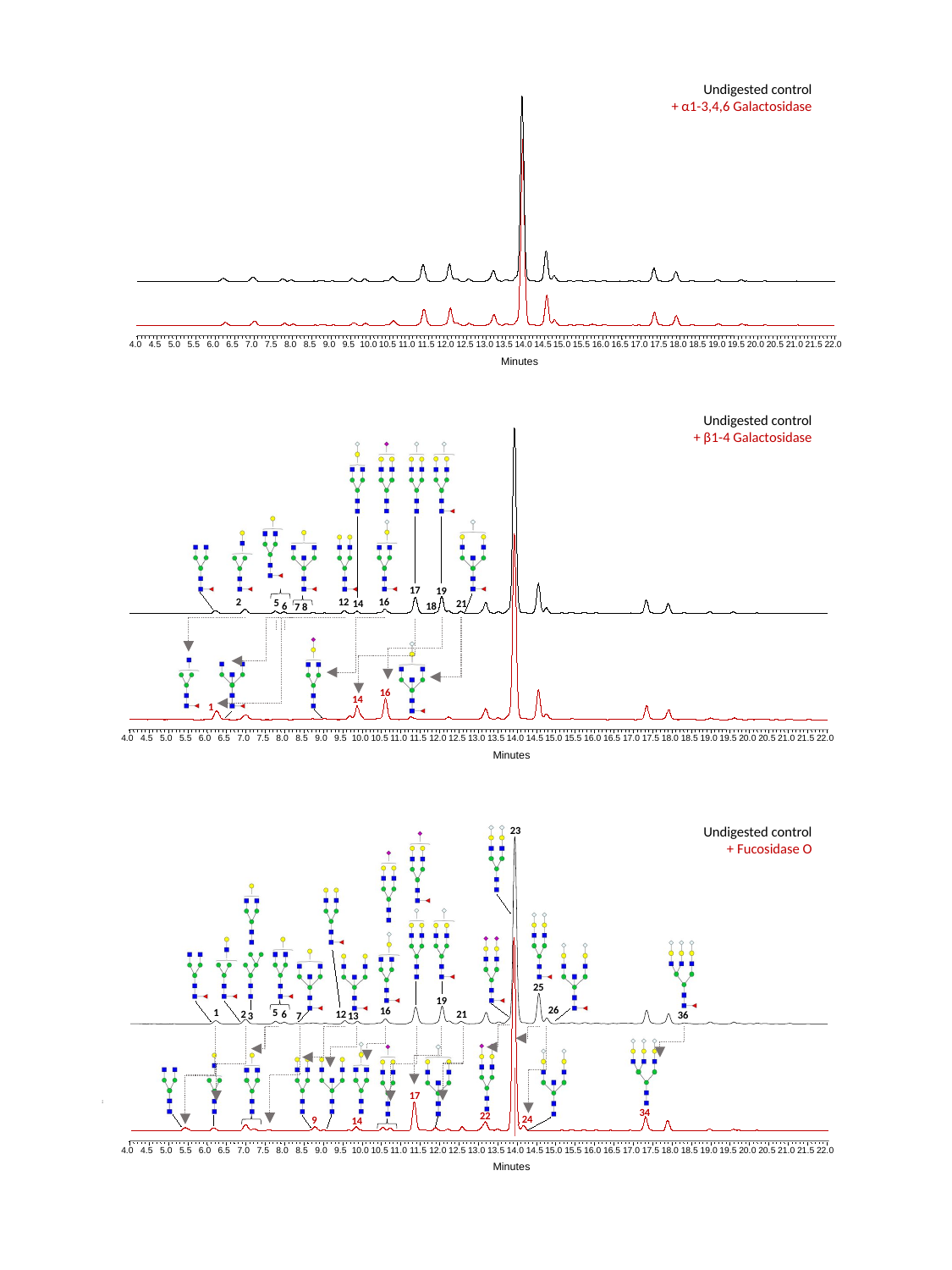

| 24 | 38 | 44 | 13,8 | 10,4 | 0,31 | 2533,0 | 1266,5 | 2432,86 | 5 | 6 | 0 | 0 | 1 | A3G3SGc1 | | Neuraminidase | | | | | | | | | | | | | | | | | | | | | | | | |
| --- | --- | --- | --- | --- | --- | --- | --- | --- | --- | --- | --- | --- | --- | --- | --- | --- | --- | --- | --- | --- | --- | --- | --- | --- | --- | --- | --- | --- | --- | --- | --- | --- | --- | --- | --- | --- | --- | --- | --- | --- |
| 24 | 38 | 44 | 13,8 | 10,4 | 0,31 | 2533,0 | 1266,5 | 2432,86 | 5 | 6 | 0 | 0 | 1 | A3G3SGc1 | | Neuraminidase | | | | | | | | | | | | | | | | | | | | | | | | |
| --- | --- | --- | --- | --- | --- | --- | --- | --- | --- | --- | --- | --- | --- | --- | --- | --- | --- | --- | --- | --- | --- | --- | --- | --- | --- | --- | --- | --- | --- | --- | --- | --- | --- | --- | --- | --- | --- | --- | --- | --- |
Undigested control
+ α1-3,4,6 Galactosidase
4.0
4.5
5.0
5.5
6.0
6.5
7.0
7.5
8.0
8.5
9.0
9.5
10.0
10.5
11.0
11.5
12.0
12.5
13.0
13.5
14.0
14.5
15.0
15.5
16.0
16.5
17.0
17.5
18.0
18.5
19.0
19.5
20.0
20.5
21.0
21.5
22.0
Minutes
Undigested control
+ β1-4 Galactosidase
17
19
2
12
16
5
14
21
18
6
7 8
16
14
1
4.0
4.5
5.0
5.5
6.0
6.5
7.0
7.5
8.0
8.5
9.0
9.5
10.0
10.5
11.0
11.5
12.0
12.5
13.0
13.5
14.0
14.5
15.0
15.5
16.0
16.5
17.0
17.5
18.0
18.5
19.0
19.5
20.0
20.5
21.0
21.5
22.0
Minutes
Undigested control
+ Fucosidase O
23
25
19
26
16
1
5
12
6
2
21
36
3
13
7
17
34
EU
22
24
9
14
4.0
4.5
5.0
5.5
6.0
6.5
7.0
7.5
8.0
8.5
9.0
9.5
10.0
10.5
11.0
11.5
12.0
12.5
13.0
13.5
14.0
14.5
15.0
15.5
16.0
16.5
17.0
17.5
18.0
18.5
19.0
19.5
20.0
20.5
21.0
21.5
22.0
Minutes

## Slide 12
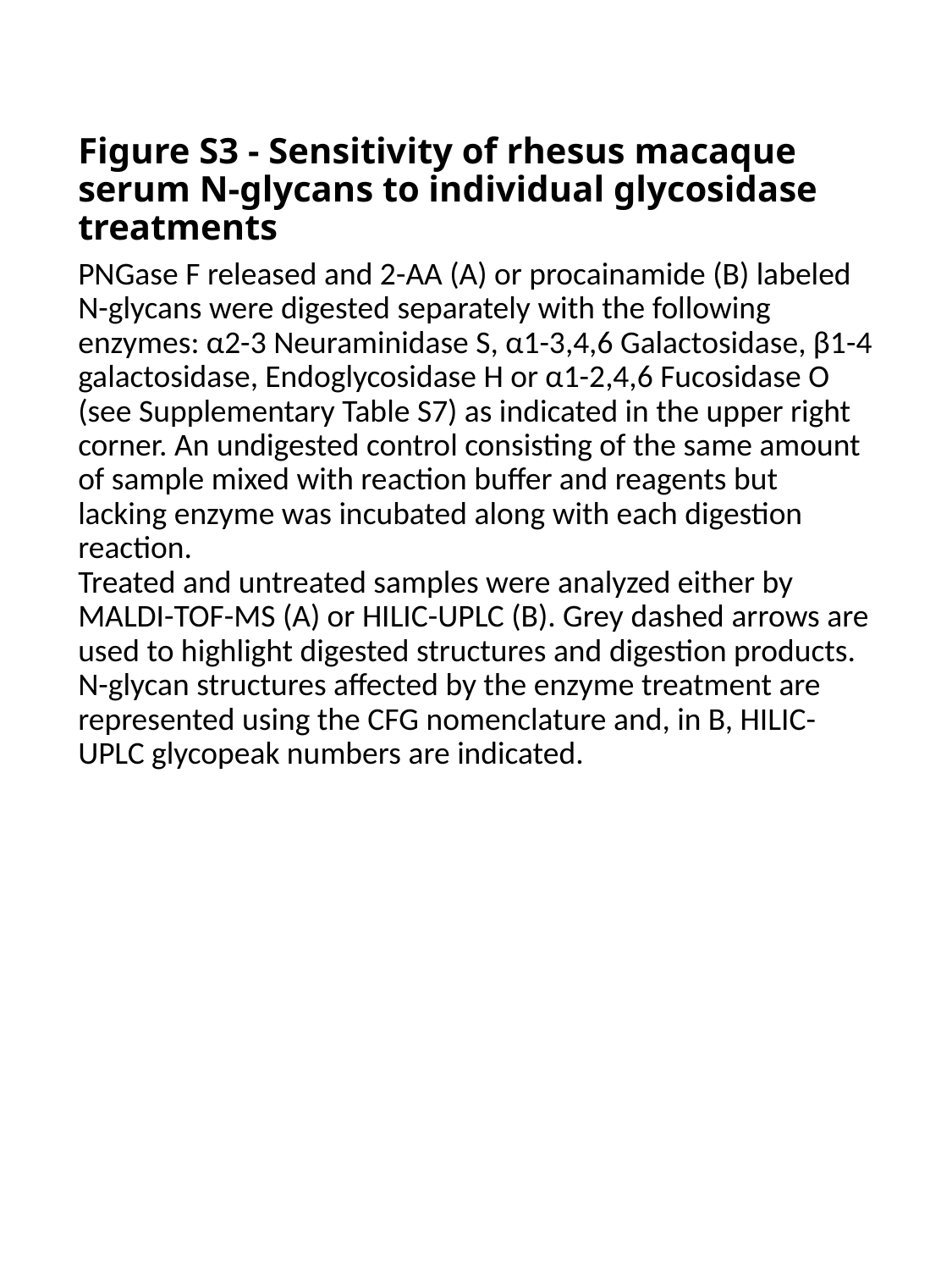

# Figure S3 - Sensitivity of rhesus macaque serum N-glycans to individual glycosidase treatments
PNGase F released and 2-AA (A) or procainamide (B) labeled N-glycans were digested separately with the following enzymes: α2-3 Neuraminidase S, α1-3,4,6 Galactosidase, β1-4 galactosidase, Endoglycosidase H or α1-2,4,6 Fucosidase O (see Supplementary Table S7) as indicated in the upper right corner. An undigested control consisting of the same amount of sample mixed with reaction buffer and reagents but lacking enzyme was incubated along with each digestion reaction.
Treated and untreated samples were analyzed either by MALDI-TOF-MS (A) or HILIC-UPLC (B). Grey dashed arrows are used to highlight digested structures and digestion products. N-glycan structures affected by the enzyme treatment are represented using the CFG nomenclature and, in B, HILIC-UPLC glycopeak numbers are indicated.

## Slide 13
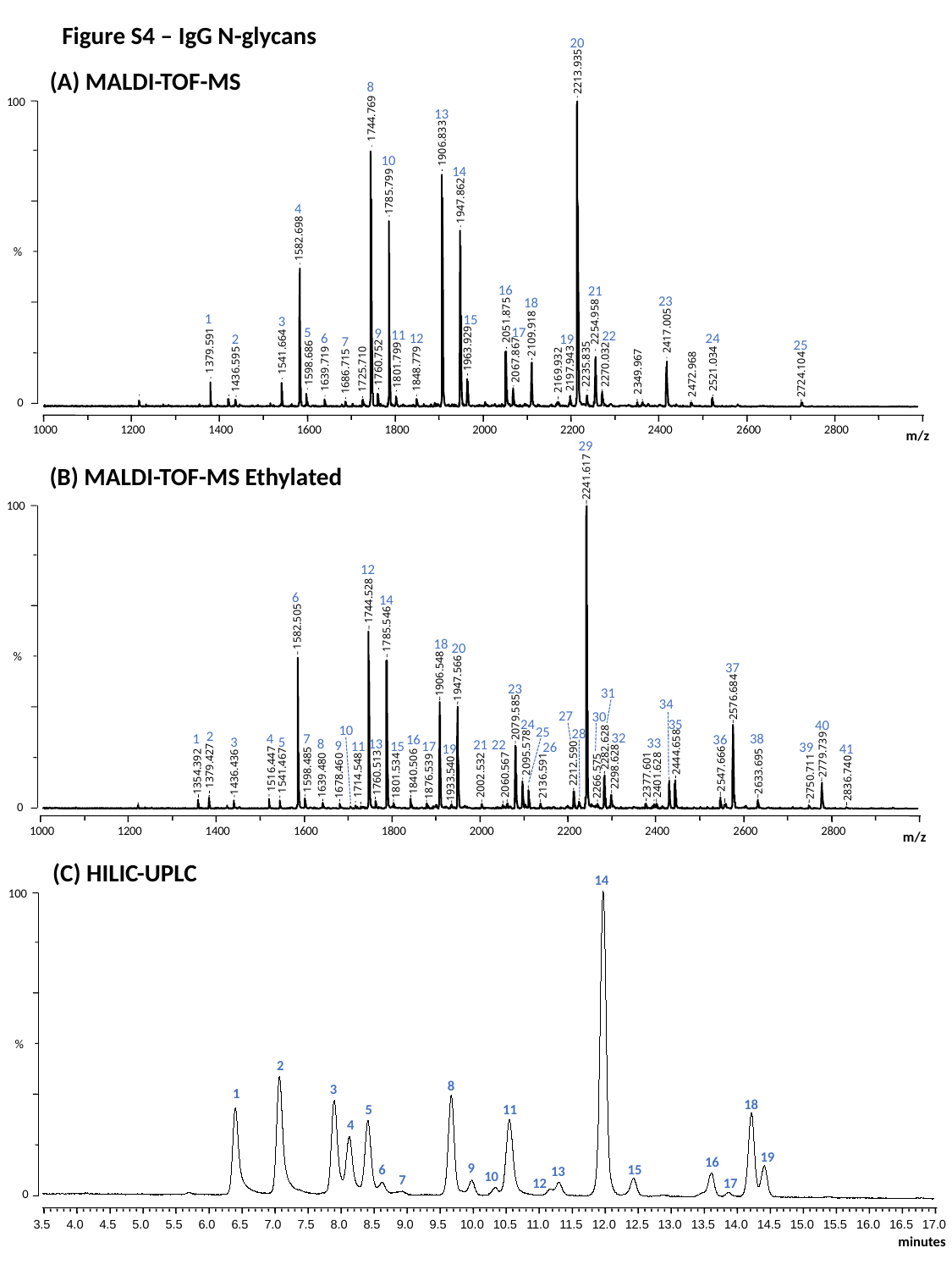

Figure S4 – IgG N-glycans
20
(A) MALDI-TOF-MS
2213.935
8
100
13
1744.769
1906.833
10
14
1785.799
4
1947.862
1582.698
%
16
21
23
18
1
15
3
2051.875
2254.958
17
5
9
11
22
6
12
24
19
2
2417.005
7
2109.918
25
1963.929
1379.591
1541.664
2067.867
1760.752
1598.686
2235.835
2270.032
1801.799
2197.943
2521.034
1848.779
1639.719
1725.710
1436.595
2169.932
1686.715
2349.967
2472.968
2724.104
0
1000
1200
1400
1600
1800
2000
2200
2400
2600
2800
m/z
29
(B) MALDI-TOF-MS Ethylated
2241.617
100
12
6
14
1744.528
1582.505
1785.546
18
20
%
37
1906.548
1947.566
23
31
34
2576.684
27
30
35
24
40
2079.585
10
25
28
2
32
4
38
1
7
36
16
5
3
33
13
8
22
21
9
15
11
17
26
39
19
41
2282.628
2444.658
2095.578
2779.739
2212.590
1379.427
2298.628
2547.666
1516.447
1598.485
1541.467
2633.695
1354.392
1840.506
1436.436
1760.513
2377.601
2060.567
2401.628
1714.548
2002.532
1639.480
1678.460
1801.534
2136.591
2266.575
1876.539
2750.711
2836.740
1933.540
0
1000
1200
1400
1600
1800
2000
2200
2400
2600
2800
m/z
(C) HILIC-UPLC
14
100
%
2
8
3
1
18
5
11
4
19
16
9
6
15
13
10
7
17
12
0
3.5
4.0
4.5
5.0
5.5
6.0
6.5
7.0
7.5
8.0
8.5
9.0
9.5
10.0
10.5
11.0
11.5
12.0
12.5
13.0
13.5
14.0
14.5
15.0
15.5
16.0
16.5
17.0
minutes

## Slide 14
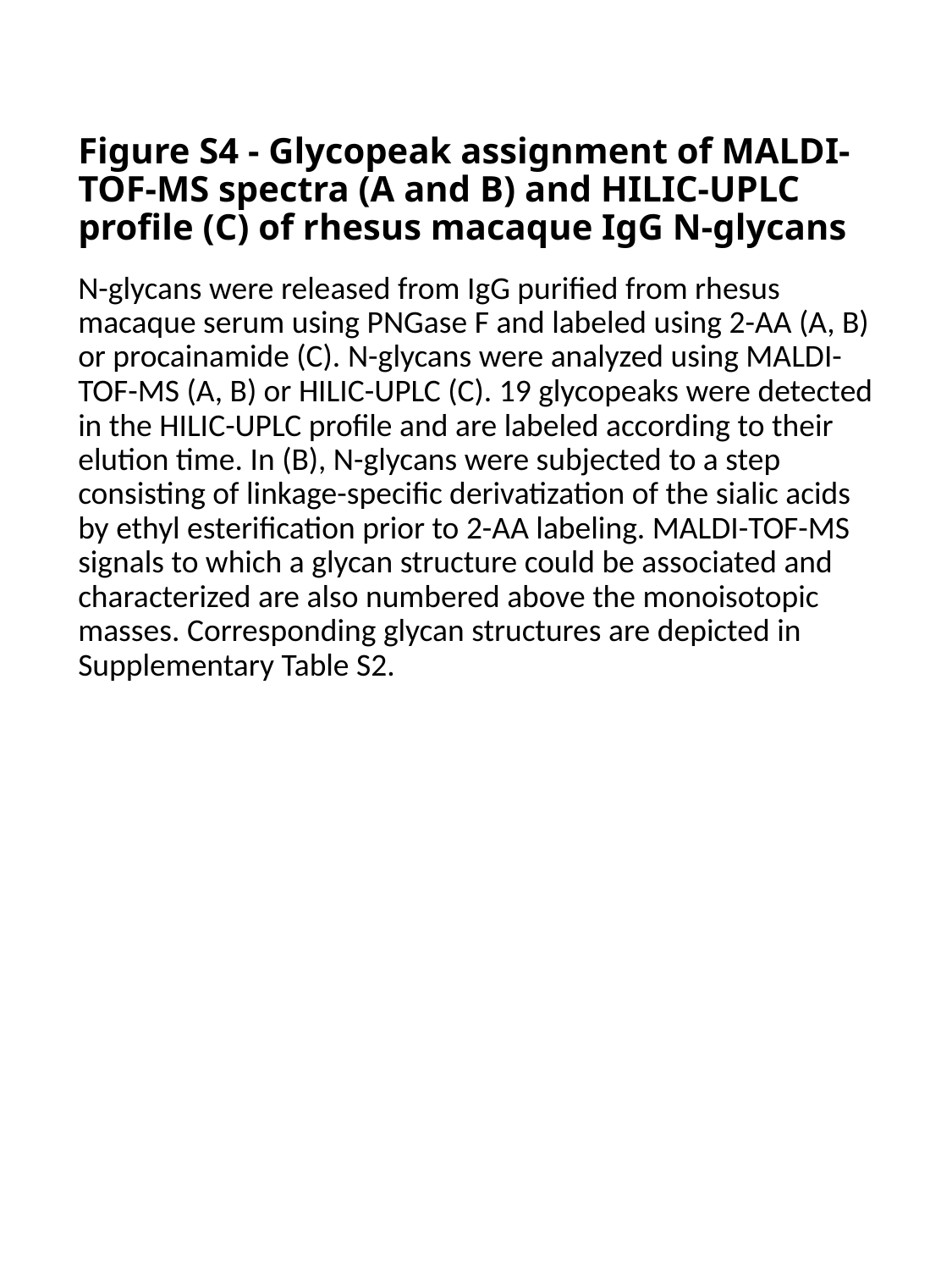

# Figure S4 - Glycopeak assignment of MALDI-TOF-MS spectra (A and B) and HILIC-UPLC profile (C) of rhesus macaque IgG N-glycans
N-glycans were released from IgG purified from rhesus macaque serum using PNGase F and labeled using 2-AA (A, B) or procainamide (C). N-glycans were analyzed using MALDI-TOF-MS (A, B) or HILIC-UPLC (C). 19 glycopeaks were detected in the HILIC-UPLC profile and are labeled according to their elution time. In (B), N-glycans were subjected to a step consisting of linkage-specific derivatization of the sialic acids by ethyl esterification prior to 2-AA labeling. MALDI-TOF-MS signals to which a glycan structure could be associated and characterized are also numbered above the monoisotopic masses. Corresponding glycan structures are depicted in Supplementary Table S2.

## Slide 15
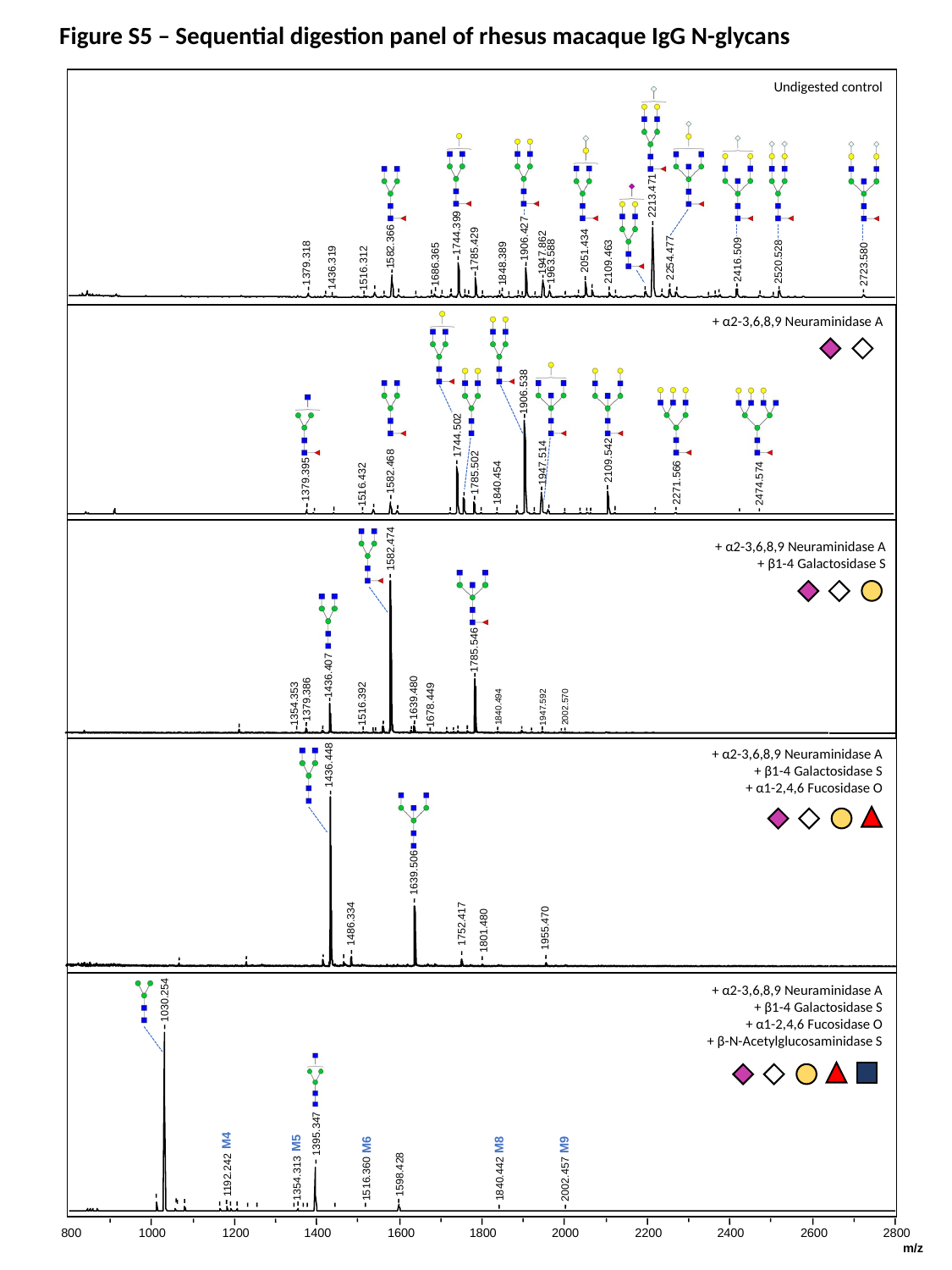

Figure S5 – Sequential digestion panel of rhesus macaque IgG N-glycans
Undigested control
2213.471
1744.399
1906.427
1582.366
1785.429
2051.434
1947.862
2254.477
2416.509
1963.588
2520.528
1379.318
2109.463
1848.389
1686.365
2723.580
1436.319
1516.312
+ α2-3,6,8,9 Neuraminidase A
1906.538
1744.502
2109.542
1947.514
1582.468
1785.502
1379.395
2271.566
1840.454
2474.574
1516.432
+ α2-3,6,8,9 Neuraminidase A
+ β1-4 Galactosidase S
1582.474
1785.546
1436.407
1639.480
1379.386
1354.353
1516.392
1678.449
2002.570
1840.494
1947.592
+ α2-3,6,8,9 Neuraminidase A
+ β1-4 Galactosidase S
+ α1-2,4,6 Fucosidase O
1436.448
1639.506
1486.334
1752.417
1955.470
1801.480
+ α2-3,6,8,9 Neuraminidase A
+ β1-4 Galactosidase S
+ α1-2,4,6 Fucosidase O
+ β-N-Acetylglucosaminidase S
1030.254
M9
M4
M5
1395.347
M8
M6
1598.428
1192.242
1354.313
1516.360
1840.442
2002.457
1400
1600
1800
2000
2200
2400
2600
2800
800
1000
1200
m/z

## Slide 16
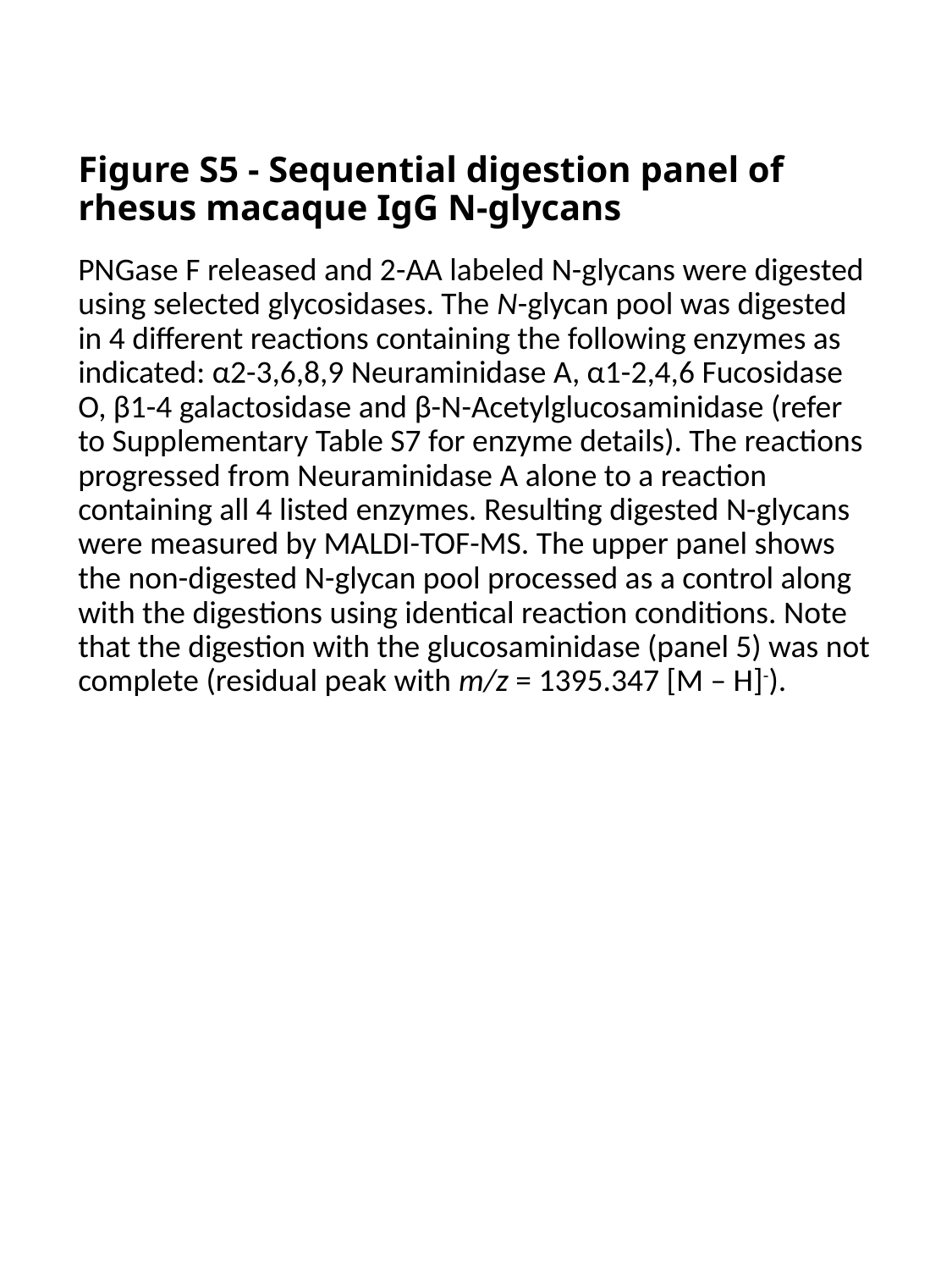

# Figure S5 - Sequential digestion panel of rhesus macaque IgG N-glycans
PNGase F released and 2-AA labeled N-glycans were digested using selected glycosidases. The N-glycan pool was digested in 4 different reactions containing the following enzymes as indicated: α2-3,6,8,9 Neuraminidase A, α1-2,4,6 Fucosidase O, β1-4 galactosidase and β-N-Acetylglucosaminidase (refer to Supplementary Table S7 for enzyme details). The reactions progressed from Neuraminidase A alone to a reaction containing all 4 listed enzymes. Resulting digested N-glycans were measured by MALDI-TOF-MS. The upper panel shows the non-digested N-glycan pool processed as a control along with the digestions using identical reaction conditions. Note that the digestion with the glucosaminidase (panel 5) was not complete (residual peak with m/z = 1395.347 [M – H]-).

## Slide 17
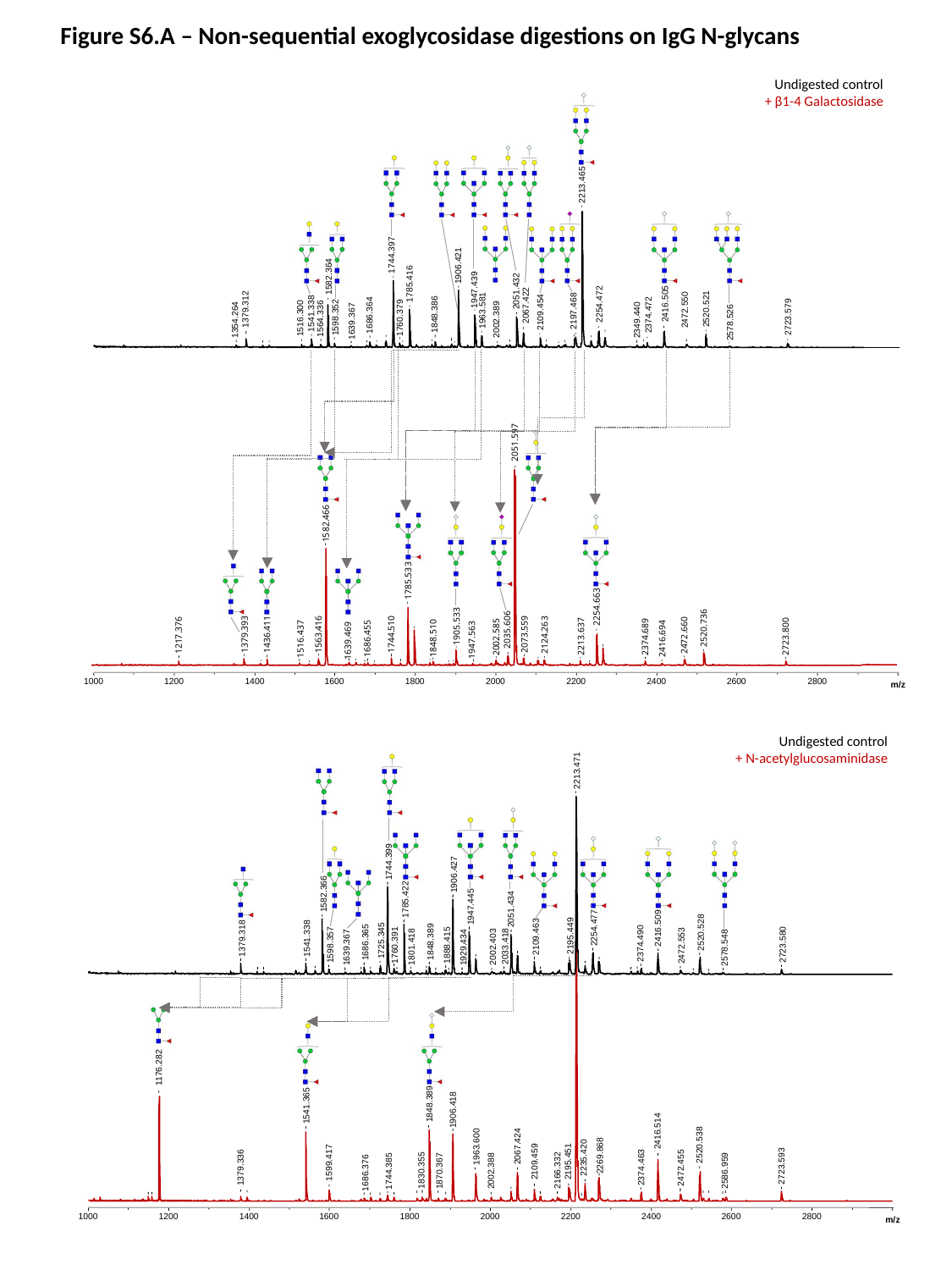

Figure S6.A – Non-sequential exoglycosidase digestions on IgG N-glycans
Undigested control
+ β1-4 Galactosidase
2213.465
1744.397
1906.421
1582.364
1785.416
1947.439
2051.432
2416.505
2254.472
2067.422
2520.521
1379.312
2472.550
1963.581
2197.468
2109.454
1541.338
1848.386
2374.472
1686.364
2723.579
1598.352
1760.379
1516.300
1564.336
2002.389
1354.264
2349.440
1639.367
2578.526
2051.597
1582.466
1785.533
2254.663
1905.533
2520.736
2035.606
2073.559
1379.393
1436.411
1563.416
1744.510
2124.263
2472.660
1217.376
2213.637
2723.800
2374.689
2002.585
1848.510
1686.455
2416.694
1516.437
1947.563
1639.469
1000
1200
1400
1600
1800
2000
2200
2400
2600
2800
m/z
Undigested control
+ N-acetylglucosaminidase
2213.471
1744.399
1906.427
1582.366
1785.422
1947.445
2051.434
2254.477
2416.509
2520.528
2195.449
2109.463
1379.318
1541.338
1725.345
1848.389
1686.365
2374.490
2723.580
1598.357
1760.391
1888.415
2472.553
2033.418
2002.403
1801.418
1639.367
1929.434
2578.548
1176.282
1848.389
1541.365
1906.418
2416.514
2520.538
2067.424
1963.600
2269.868
2235.420
2109.459
2195.451
1599.417
2723.593
2374.463
1379.336
2472.455
1830.355
2166.332
2586.959
1870.367
2002.388
1744.385
1686.376
1000
1200
1400
1600
1800
2000
2200
2400
2600
2800
m/z

## Slide 18
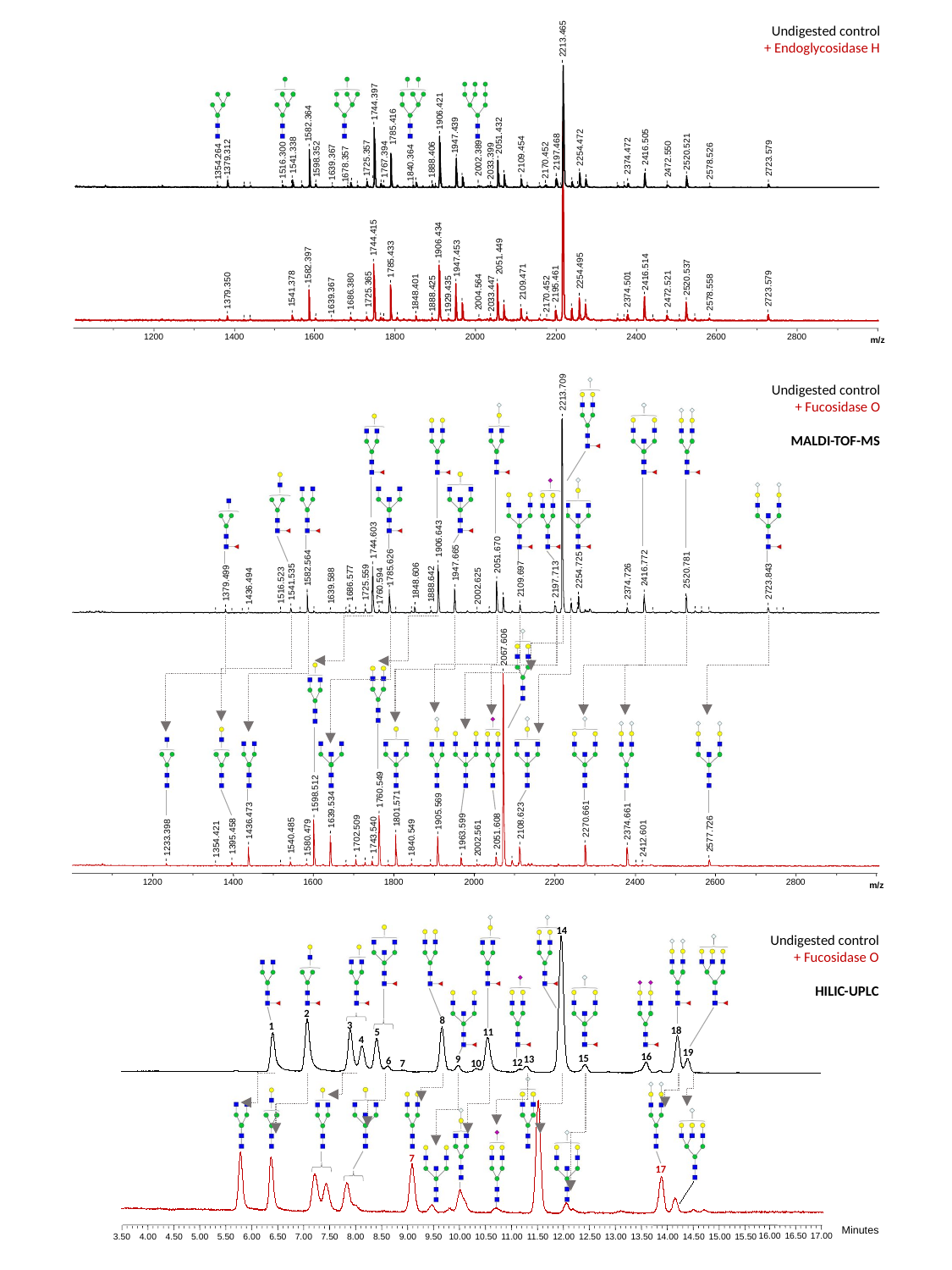

Undigested control
+ Endoglycosidase H
2213.465
1744.397
1906.421
1582.364
1785.416
1947.439
2051.432
2416.505
2254.472
2520.521
2197.468
2109.454
1541.338
2374.472
1379.312
2002.389
1725.357
2723.579
2472.550
1598.352
1767.394
1888.406
2170.452
1516.300
2578.526
2033.399
1354.264
1639.367
1840.364
1678.357
1744.415
1906.434
2051.449
1947.453
1785.433
1582.397
2254.495
2416.514
2520.537
2109.471
2195.461
2723.579
1541.378
2472.521
2374.501
1725.365
1379.350
1686.380
2004.564
1848.401
2578.558
2033.447
1888.425
1929.435
2170.452
1639.367
1200
1400
1600
1800
2000
2200
2400
2600
2800
m/z
Undigested control
+ Fucosidase O
MALDI-TOF-MS
2213.709
1906.643
1744.603
2051.670
1947.665
1785.626
1582.564
2416.772
2520.781
2254.725
2109.697
2197.713
1848.606
2374.726
2723.843
1541.535
1725.559
1686.577
1379.499
1888.642
1516.523
1639.588
1436.494
2002.625
1760.594
2067.606
1760.549
1598.512
1801.571
1639.534
1905.569
2270.661
1436.473
2108.623
2374.661
2051.608
1963.599
1702.509
2577.726
1743.540
1540.485
1395.458
1233.398
1580.479
1840.549
2002.561
2412.601
1354.421
1200
1400
1600
1800
2000
2200
2400
2600
2800
m/z
14
Undigested control
+ Fucosidase O
HILIC-UPLC
2
8
3
1
18
5
11
4
19
16
15
9
13
6
12
10
7
 7
17
Minutes
16.00
16.50
17.00
3.50
4.00
4.50
5.00
5.50
6.00
6.50
7.00
7.50
8.00
8.50
9.00
9.50
10.00
10.50
11.00
11.50
12.00
12.50
13.00
13.50
14.00
14.50
15.00
15.50

## Slide 19
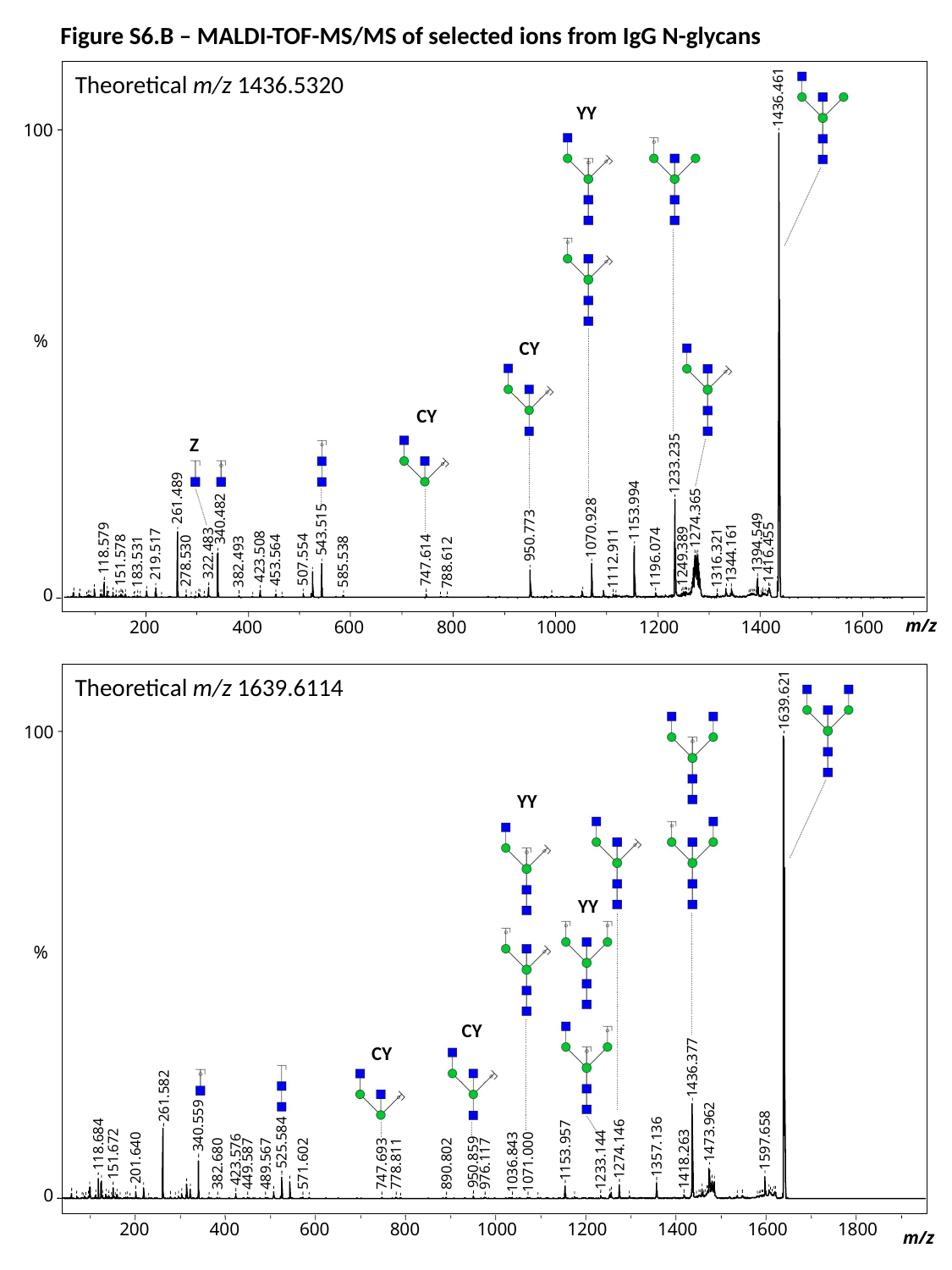

Figure S6.B – MALDI-TOF-MS/MS of selected ions from IgG N-glycans
Theoretical m/z 1436.5320
1436.461
YY
100
%
CY
CY
Z
1233.235
261.489
1153.994
1274.365
340.482
1070.928
543.515
950.773
1394.549
118.579
1416.455
1344.161
322.483
219.517
1249.389
423.508
1196.074
1316.321
1112.911
507.554
747.614
151.578
453.564
278.530
183.531
382.493
585.538
788.612
0
m/z
200
400
600
800
1000
1200
1400
1600
Theoretical m/z 1639.6114
1639.621
100
YY
YY
%
CY
CY
1436.377
261.582
340.559
1473.962
1597.658
525.584
118.684
1357.136
1274.146
1153.957
151.672
201.640
1418.263
423.576
1233.144
1036.843
1071.000
950.859
382.680
449.587
489.567
571.602
747.693
778.811
890.802
976.117
0
200
400
600
800
1000
1200
1400
1600
1800
m/z

## Slide 20
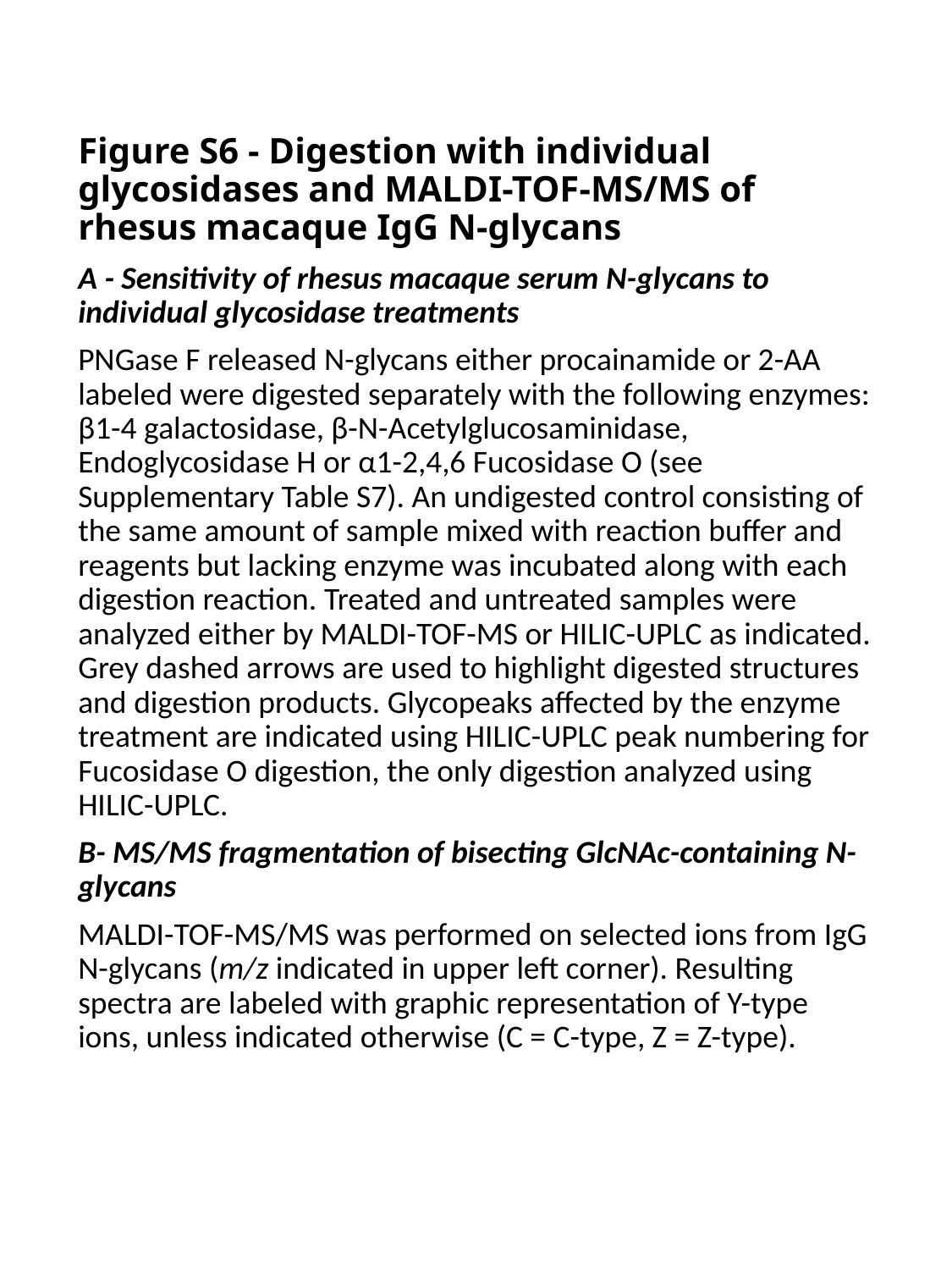

# Figure S6 - Digestion with individual glycosidases and MALDI-TOF-MS/MS of rhesus macaque IgG N-glycans
A - Sensitivity of rhesus macaque serum N-glycans to individual glycosidase treatments
PNGase F released N-glycans either procainamide or 2-AA labeled were digested separately with the following enzymes: β1-4 galactosidase, β-N-Acetylglucosaminidase, Endoglycosidase H or α1-2,4,6 Fucosidase O (see Supplementary Table S7). An undigested control consisting of the same amount of sample mixed with reaction buffer and reagents but lacking enzyme was incubated along with each digestion reaction. Treated and untreated samples were analyzed either by MALDI-TOF-MS or HILIC-UPLC as indicated. Grey dashed arrows are used to highlight digested structures and digestion products. Glycopeaks affected by the enzyme treatment are indicated using HILIC-UPLC peak numbering for Fucosidase O digestion, the only digestion analyzed using HILIC-UPLC.
B- MS/MS fragmentation of bisecting GlcNAc-containing N-glycans
MALDI-TOF-MS/MS was performed on selected ions from IgG N-glycans (m/z indicated in upper left corner). Resulting spectra are labeled with graphic representation of Y-type ions, unless indicated otherwise (C = C-type, Z = Z-type).

## Slide 21
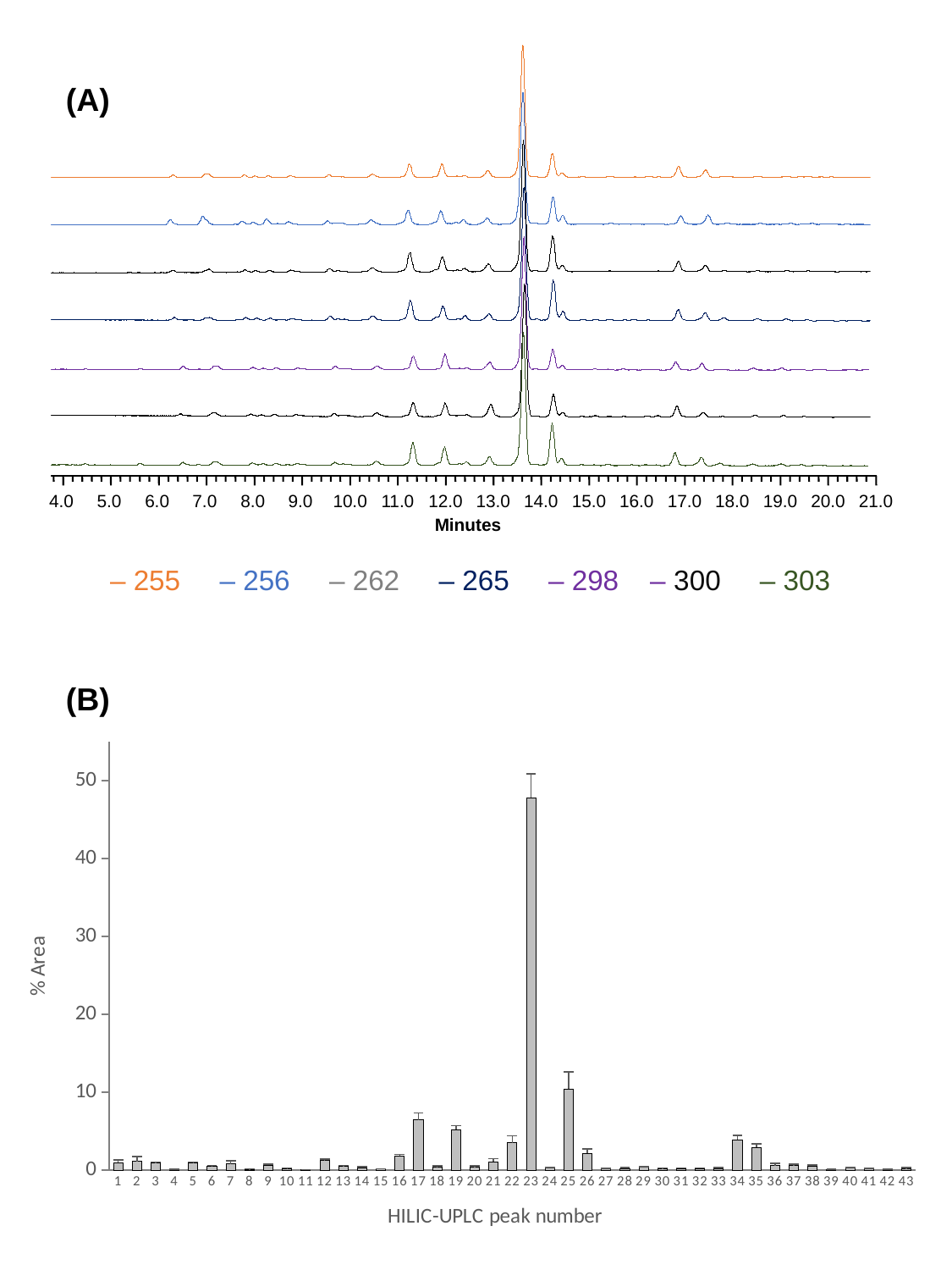

(A)
4.0
5.0
6.0
7.0
8.0
9.0
10.0
11.0
12.0
13.0
14.0
15.0
16.0
17.0
18.0
19.0
20.0
21.0
Minutes
– 255 – 256 – 262 – 265 – 298 – 300 – 303
(B)
### Chart
| Category | |
|---|---|
| 1 | 1.015 |
| 2 | 1.159285714285714 |
| 3 | 0.9242857142857143 |
| 4 | 0.10214285714285716 |
| 5 | 0.937142857142857 |
| 6 | 0.5657142857142857 |
| 7 | 0.8021428571428572 |
| 8 | 0.0935714285714286 |
| 9 | 0.6628571428571429 |
| 10 | 0.24285714285714294 |
| 11 | 0.06500000000000002 |
| 12 | 1.26 |
| 13 | 0.5814285714285715 |
| 14 | 0.3607142857142857 |
| 15 | 0.1542857142857143 |
| 16 | 1.8764285714285713 |
| 17 | 6.471428571428572 |
| 18 | 0.37928571428571434 |
| 19 | 5.2092857142857145 |
| 20 | 0.3950000000000001 |
| 21 | 1.0857142857142859 |
| 22 | 3.523571428571429 |
| 23 | 47.82714285714285 |
| 24 | 0.3085714285714286 |
| 25 | 10.43214285714286 |
| 26 | 2.205714285714286 |
| 27 | 0.215 |
| 28 | 0.24571428571428575 |
| 29 | 0.39714285714285713 |
| 30 | 0.1942857142857143 |
| 31 | 0.2178571428571429 |
| 32 | 0.2564285714285714 |
| 33 | 0.2064285714285714 |
| 34 | 3.934285714285714 |
| 35 | 2.9457142857142857 |
| 36 | 0.5907142857142856 |
| 37 | 0.6085714285714285 |
| 38 | 0.535 |
| 39 | 0.15 |
| 40 | 0.3178571428571429 |
| 41 | 0.1971428571428572 |
| 42 | 0.14285714285714285 |
| 43 | 0.2007142857142857 |

## Slide 22
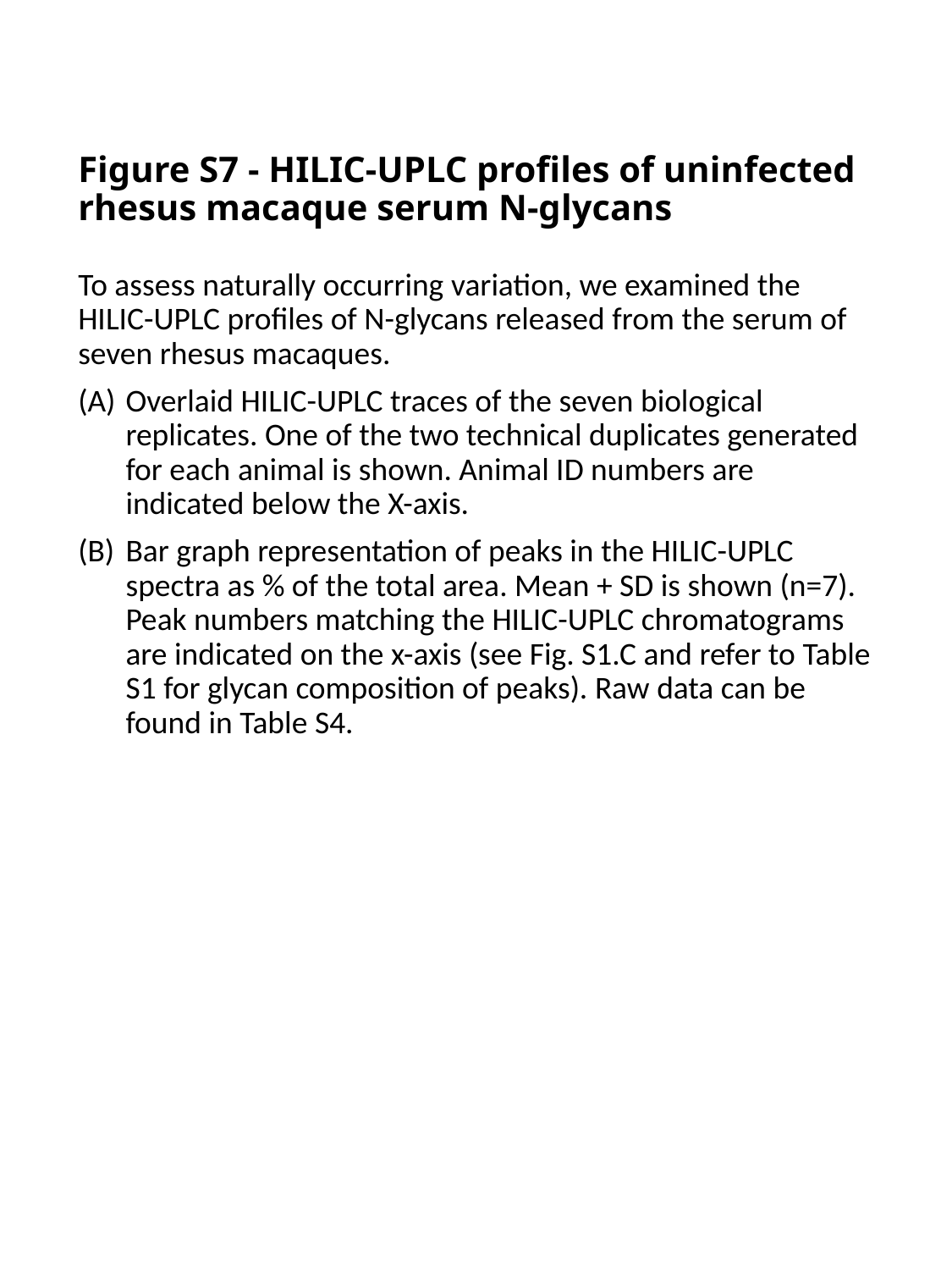

# Figure S7 - HILIC-UPLC profiles of uninfected rhesus macaque serum N-glycans
To assess naturally occurring variation, we examined the HILIC-UPLC profiles of N-glycans released from the serum of seven rhesus macaques.
Overlaid HILIC-UPLC traces of the seven biological replicates. One of the two technical duplicates generated for each animal is shown. Animal ID numbers are indicated below the X-axis.
Bar graph representation of peaks in the HILIC-UPLC spectra as % of the total area. Mean + SD is shown (n=7). Peak numbers matching the HILIC-UPLC chromatograms are indicated on the x-axis (see Fig. S1.C and refer to Table S1 for glycan composition of peaks). Raw data can be found in Table S4.
